# Acknowledging Individual Responsibility while Emphasizing Social Determinants in Narratives to Promote Obesity-Reducing Public Policy: A Randomized Experiment

**DOI:** 10.1371/journal.pone.0117565

**Published:** 2015-02-23

**Authors:** Jeff Niederdeppe, Sungjong Roh, Michael A. Shapiro

**Affiliations:** Department of Communication, Cornell University, Ithaca, New York, United States of America; University of Bath, UNITED KINGDOM

## Abstract

This study tests whether policy narratives designed to increase support for obesity-reducing public policies should explicitly acknowledge individual responsibility while emphasizing social, physical, and economic (social) determinants of obesity. We use a web-based, randomized experiment with a nationally representative sample of American adults (n = 718) to test hypotheses derived from theory and research on narrative persuasion. Respondents exposed to narratives that acknowledged individual responsibility while emphasizing obesity’s social determinants were less likely to engage in counterargument and felt more empathy for the story’s main character than those exposed to a message that did not acknowledge individual responsibility. Counterarguing and affective empathy fully mediated the relationship between message condition and support for policies to reduce rates of obesity. Failure to acknowledge individual responsibility in narratives emphasizing social determinants of obesity may undermine the persuasiveness of policy narratives. Omitting information about individual responsibility, a strongly-held American value, invites the public to engage in counterargument about the narratives and reduces feelings of empathy for a character that experiences the challenges and benefits of social determinants of obesity.

## Introduction

Rates of obesity in the U.S. have increased over the past 25 years, such that more than one in three Americans (35.7 percent) are now obese [1]. While genetic predispositions, lifestyle decisions, and the social, physical, and economic environment (social determinants) influence rates of obesity, social determinants have had the largest role in driving the epidemic [2]. Reducing obesity rates will require a broad set of public policies to address these social determinants, but there is limited public support for such policies [3]. This has led researchers and policy experts to develop and test narratives designed to increase public support for policies that target obesity’s social causes. This study tests whether narratives designed to increase support for obesity policy should acknowledge individual responsibility for achieving a healthy weight. We use a web-based, randomized experiment with a nationally representative U.S. sample to test hypotheses derived from research on narrative persuasion on the impact of stories about obesity.

### Obesity policy narratives and individual responsibility

Policy narratives play a significant role in contemporary policy debates and, more recently, have been the focus of quantitative policy studies [4, 5]. Policy narratives feature a plot, setting, and characters (heroes, villains, and/or victims). These narratives highlight the causes of social problems and emphasize the potential effects of policies designed to address them [4–6]. Recent policy narrative studies offer evidence of their impact on public opinion [7–9].

In the context of obesity policy narratives, several authors argue that it is essential to acknowledge individual responsibility when describing obesity’s social determinants [10, 11], because failure to acknowledge the strongly-held value of rugged individualism embedded in American culture will lead to a backlash, particularly among political conservatives [12].

At the same time, studies testing the efficacy of this strategy have shown mixed results.

One study found that a short vignette explicitly acknowledging individual responsibility, while emphasizing social determinants of health, reduced the likelihood of counterarguing among Republicans [13]. Another effort, however, found greater support for obesity-reducing policies among political conservatives in response to a short narrative (vs. no message) that avoided reference to individual responsibility while emphasizing social determinants [8]. These authors were cautious about this finding, however, due to the use of a convenience sample and the lack of a theoretical mechanism to explain the pattern of results.

These mixed results highlight the importance of identifying when acknowledging individual responsibility in narratives about the broader causes of obesity may influence support for efforts to address social determinants. The current study offers the benefits of a nationally representative sample and the ability to test theoretical explanations for differences in policy support in response to narratives that acknowledge (or not) individual responsibility when describing obesity’s social causes.

### Pathways to policy narrative persuasion

We propose a set of expected public responses that imply both persuasive advantages and disadvantages for narratives that acknowledge individual responsibility while emphasizing social determinants of obesity, compared to narratives that avoid mention of individual responsibility. Specifically, we modeled the persuasive effects of policy narratives by taking into account their impacts on cognitive responses and character (here, victims of obesity) perceptions.

One recent analysis found that a short story strongly acknowledging individual responsibility while emphasizing obesity’s social causes increased the likelihood of respondents engaging in counterelaboration (i.e., thoughts focused exclusively on individual causes or solutions for obesity, without refuting external ones) [8]. While a follow-up study did not replicate this result, these authors did find that another story strongly acknowledging individual responsibility produced less simple elaboration (i.e., thoughts focused only on social causes or societal solutions for obesity) than a narrative that avoided reference to individual responsibility [14]. Combined, these studies suggest that story content about individual responsibility can focus audience thoughts on that content (increasing counterelaboration) at the expense of thoughts about social causes or solutions (reducing simple elaboration). We thus predict that a story strongly acknowledging individual responsibility will produce less simple elaboration (Hypothesis 1; H1) and more counter-elaboration (H2) than a story that does not acknowledge it.

Individual responsibility is a strongly held American value [12], and individuals are often mentioned first in response to questions about who is responsible for causing and addressing obesity [15]. Respondents seem likely to question the omission of strongly held and accessible beliefs about individual responsibility in a message about obesity’s causes [13]. We thus predict that a story acknowledging individual responsibility will produce less counter-arguing (i.e., thoughts that explicitly refute the intended persuasive theme that broader societal forces cause or bear responsibility for addressing obesity) than a story that does not acknowledge it (H3).

Affective Disposition Theory argues that morality judgments shape audience members’ feelings toward story characters [16]. Audiences want good things to happen to characters that demonstrate strong moral character. Since Americans consider individual responsibility both a value and moral virtue [12], readers of a narrative would appear likely to have more favorable judgments of a character that demonstrates strong individual responsibility than a responsibility without this virtue [8]. These favorable judgments, in turn, should increase the extent to which readers see themselves as similar to a character and feel for that character and her situation [16]. We thus predict that a story acknowledging individual responsibility will produce greater perceived similarity (H4) and more empathy (H5) than a story that does not acknowledge it.

### Effects on support for obesity policy

To summarize, predictions about two cognitive responses (simple elaboration and counter-elaboration) suggest a persuasive advantage for a story that avoids mention of individual responsibility while emphasizing social determinants of obesity. Predictions about another cognitive response (counterarguing) and both character perceptions (perceived similarity and empathy), however, suggest a persuasive advantage for a story that acknowledges individual responsibility. On balance, these lead to a prediction that a story acknowledging individual responsibility (and emphasizing social causes) will produce more support for policies to reduce rates of obesity than a story that does not acknowledge individual responsibility (H6). We further suggest that this relationship will be mediated by reduced counterarguing (H7), increased perceived similarity (H8), and increased empathy (H9).

In a representative democracy like the US where no single party is likely to hold majority support, it will be important to generate support for obesity policy across partisan lines. However, previous studies have produced mixed findings about the degree to which political ideology and political party affiliation shape responses to messages about obesity-related policies. [8, 12–13] We thus examine whether narrative design effects (acknowledging individual responsibility or not) on support for obesity policy differ by respondents’ political partisanship (Research Question 1; RQ1).

## Methods

### Sample recruitment

We report on a web-based, randomized experiment between July 28^th^ and August 11^th^, 2011 conducted among a nationally representative panel of US adults maintained by GfK Knowledge Networks (GfK) and recruited via probability sampling. GfK panelists are recruited by address-based random sampling, covering 97% of American households and resulting in distributions that approximate US Census results on most major demographic categories (S1 Table). If respondents do not already have home internet access, GfK provides them with a computer and internet access when they agree to participate in the panel. Due to the use of address-based sampling, the panel includes households with listed and unlisted numbers as well as cell phone-only households. Those who agree to participate in the panel complete a demographic profile and then respond periodically to web-based surveys. GfK’s panel recruitment rate was 21 percent at the time of the study. GfK invited a random sample of 1,462 panelists to participate in the study. 718 participants actively consented using an electronic form and completed the study, producing a cooperation rate of 49 percent and a response rate (recruitment x cooperation) of 10 percent. A member of the Office of Research Integrity and Assurance (ORIA) at Cornell University reviewed this project and found it to qualify for Exemption from institutional review board (IRB) review according to paragraph #2 of the Department of Health and Human Services Code of Federal Regulations 45 CFR 46.101(b) on May 2nd, 2011 (protocol ID 1104002183).

### Stimuli

We randomly assigned participants to read one of nine versions of the survey using a 2 (acknowledging individual responsibility or not) by 2 (a Republican or Democratic partisan cue which appeared halfway through the story) by 2 (asking respondents to read the story with empathy or rationality and detachment) factorial design with an additional no-exposure control group. Respondents in non-control conditions read a 1-page story about Michele who struggled with her weight but benefitted from a community intervention to improve her neighborhood. Each story emphasized the high cost and lack of access to healthy foods, widespread availability of unhealthy foods, time constraints from a low-income job, and a lack of safe and affordable places for exercise in Michele’s neighborhood. Each story further described efforts to add a local supermarket, bicycle trails, and walking paths (see S1 Survey Codebook attachment for full scripts).

Conditions that acknowledged individual responsibility conveyed Michele’s sense of responsibility for losing weight and getting healthy throughout the narrative (e.g., “Michele has always believed that it is her own personal responsibility to be healthy, but it hasn’t been easy”). Conditions that did not acknowledge individual responsibility were identical, except that they offered no indication that Michele took responsibility for her own health or weight loss (e.g., “Many people like Michele don’t have the time or energy to adopt major lifestyle changes”).

### Participants and manipulation checks

There were no differences in demographics between the control, no individual responsibility, and high individual responsibility conditions (S1 Table). While GfK calculates statistical weights in an effort to reflect the national population, the analyses reported in this paper did not use them. We made this decision for three reasons: (1) the substantive findings were similar regardless of whether or not weights were applied, (2) in all analyses we controlled for the demographic factors that were used to create the weights, which serves to adjust for possible confounding of these variables, and (3) recommended procedures for formally testing statistical mediation, a key step in the analysis do not permit the use of sampling weights [17].

Immediately after exposure, we compared narratives that acknowledged (or not) individual responsibility on two measures: perceived emphasis on individual responsibility (3 items, 5-point Likert scale, α = .85; e.g., “This story suggested that Michele is personally responsible for losing weight”) and perceived emphasis on societal responsibility (2 items, same scale, *r* = .32; “This story suggested that society is responsible for helping Michele to lose weight”). Respondents perceived greater emphasis on individual responsibility in conditions that acknowledged individual responsibility (IR) than in conditions that did not acknowledge individual responsibility (NoIR) (*M*_IR_ = 3.8, *M*_NoIR_ = 2.6), *t* (639) = 18.2, *p* <. 001. The individual responsibility condition was greater than the midpoint (3) of the individual responsibility emphasis scale (*p* <. 001, 1-sample t-test) while the no individual responsibility condition was not (*p* = .99). Participants perceived more emphasis on societal responsibility in the no individual responsibility condition (*M*_IR_ = 3.4, *M*_NoIR_ = 3.6), *t* (634) = −3.9, *p* <. 001, but both values were higher than the midpoint (3) of the societal responsibility emphasis scale (*ps* <. 001, 1-sample t-test). The individual responsibility manipulation was thus deemed successful.

The empathy and partisan cue manipulations were much shorter, subtler, and designed as exploratory. The empathy manipulation occurred prior to reading the story. In the high empathy condition, we asked respondents “to try to imagine how Michele feels about what has happened and how it has affected her life. Try to feel the full impact of what Michele has been through and how she feels as a result.” In the low empathy condition, we asked respondents “to try to take an objective perspective toward what is described. Try not to get caught up in how Michele feels; just remain objective and detached.” We compared levels of affective empathy (described below) between conditions that asked respondents to read the message with empathy and those that asked respondents to read with detachment. Conditions did not differ on this measure, described in the methods section (*M*_empathy_ = 2.5, *M*_objective_ = 2.4), *t* (627) = 1.6, *p* >. 05, 1-tailed.

The partisan cue manipulation was embedded about one-third of the way through the narrative, in the middle of the third paragraph. The passage, coming from the perspective of Michele and explaining her general lack of free time, was worded as follows: “With two jobs and the time I spend volunteering for the local committee for the [Democratic/Republican] Party…” We tested whether Republicans and Democrats perceived themselves as more similar to Michelle in the condition where she volunteered for the matched political party (Republican Party (RP) for Republicans; Democratic Party (DP) for Democrats. This was not true for either group (*M*_RP_ = 2.5, *M*_DP_ = 2.4), *t* (170) = 0.2, *p* >. 05, 1-tailed; (*M*_DP_ = 2.6, *M*_RP_ = 2.5), *t* (227) = 0.6, *p* >. 05, 1-tailed. Since these manipulation checks revealed that differences were not perceived as intended, we focused the analysis on effects of the individual responsibility manipulation.

### Dependent variable measures

We asked participants to type three thoughts that occurred to them immediately after they read the story [18]. Coders classified each thought (*N* = 2,154) along three dimensions: whether or not they contained (a) internal attributions (thoughts focused on individual, controllable (not genetic) causes or solutions for obesity), (b) external/social attributions (thoughts focused on causes or solutions for obesity external to the individual), and (c) counterarguments (thoughts that directly refuted external/social attributions). We double-coded each thought (Krippendorf’s α_internal_ = .77; α_external_ = .82; α_counter_ = .69) and resolved disagreements by consensus. We used these codes to create four mutually exclusive categories, based on categories developed in previously published research, for each thought: (a) counterarguing (≥ 1 counterargument); (b) complex integration (≥ 1 thought combining external and internal attributions and without counterarguing external attributions); (c) simple elaboration (≥ 1 thought exclusively about external attributions without counterarguing external attributions); and (d) counter-elaboration (≥ 1 thought exclusively about internal attributions without counterarguing external attributions) [14]. Each respondent could have up to 3 thoughts in up to 3 categories. Overall, more respondents had at least one thought coded as counterelaboration (57%, *n* = 407) and simple elaboration (50%, *n* = 362) than counterarguing (19%, *n* = 134) or complex integration (24%, *n* = 169). Complex integration did not differ by condition and is not considered further.

Six items gauged perceived similarity to the character (on a 5-point Likert scale, strongly disagree, 1, to strongly agree, 5; e.g., “Michelle has values that are like the values I practice”). We averaged these items into a scale (*α* = .95, *M* = 2.48, *SD* = .94).

Five previously-validated items measured affective empathy toward the character [19, 20]. All items were measured on the same 5-point scale used to gauge perceived similarity. We averaged these items into a 5-item scale (e.g., “I felt upset for those who suffer from the problem described in the message,” *α* = .83, *M* = 2.48, *SD* = .73).

We asked respondents about their degree of support (on a 5-point scale from strongly oppose, 1, to strongly support, 5) for 8 randomly ordered policies that target the social, economic, and/or physical environment to reduce obesity rates and have been proposed in recent years (e.g., “Have zoning laws requiring that all new residential and commercial developments include sidewalks and other safe paths to encourage physical activity”) [21]. We averaged them into a scale (*α* = .84; *M* = 3.46, *SD* = .75).

We gauged party identification by asking, “Generally speaking, do you consider yourself to be a Republican, a Democrat, an Independent, or Something Else?”

## Results

We conducted all study analyses using STATA v13, with the exception of the bootstrap mediation analysis, which is only available through an SPSS macro. We report unstandardized coefficients for logistic and OLS regression models shown in tables; we also report standardized OLS regression coefficients for message condition variables in the text only. Tests of H1 through H5 (using logistic or OLS regression with demographic controls) are shown in S2 Table and S3 Table. H1 was not supported (*p* >. 10); there were no differences in the likelihood of simple elaboration between the high and no individual responsibility conditions. H2 through H5 received support. Respondents were more likely to engage in counterelaboration (supporting H2, *p* <. 001), less likely to engage in counterarguing (supporting H3, *p* <. 001), perceived themselves as more similar to Michele (supporting H4, *p* <. 001, β = .29) and felt more empathy for Michele (supporting H5, *p* <. 001, β = .21) in the high individual responsibility condition compared to the no individual responsibility condition.

The formal test of H6 is presented in the first column of S4 Table. Supporting H6, those in the high individual responsibility condition were more likely to support policies to reduce rates of obesity than those in the no individual responsibility condition (*p* = .03, β = .09). The final column of S3 Table, however, reveals that respondents in neither the no individual responsibility condition (*p* = .11, β = −.10) nor the high individual responsibility condition (*p* = .76, β = −.02) had lower levels of obesity policy support than the no-exposure control group.

S4 Table also describes results of OLS models testing H7 through H9 (omitting variables did not differ by randomized condition). These models support H7 and H9, but not H8. Respondents who engaged in counterarguing had lower levels of policy support than those who did not offer a counterargument (consistent with H7; *p* = .003, β = −.11). Higher levels of affective empathy were also associated with higher levels of policy support (consistent with H9; *p* <. 001. β = .30). Counterelaboration (not hypothesized; *p* = .11, β = .06) and perceived similarity (rejecting H8; *p* = .59, β = −.02) were not associated with policy support and thus cannot be mediators [22]. The coefficient for the high versus no individual responsibility condition changed from 0.13 (*p* = .03, β = .09) to 0.01 (*p* = .93, β = .00) when both mediators were added to the model, consistent with full mediation.

We conducted formal tests of mediation using bootstrap methods with the INDIRECT macro for SPSS that estimates the overall indirect (mediated) and remaining direct effects (after accounting for mediators) of randomized condition on support for public policies to reduce rates of obesity [22]. Using a 95% confidence interval (CI) and 5,000 bootstrap resamples, CIs for indirect effects of counter-arguing (H7;. 01 to. 05) and affective empathy (H9;. 06 to. 15) did not include zero, providing full support of H7 and H9. In contrast, the CIs for indirect effects of counter-elaboration (−.001 to. 03) and perceived similarity (H8; −.05 to. 03) included zero and thus are not significant mediators (rejecting H8). Consistent with full mediation, the total indirect effect of message condition on policy support via the mediators was significant (B = .13, *p* = .03) while the remaining direct effect of message condition on support was not (B = .01, *p* = .93).

While testing relationships between respondent demographics (which were included primarily for the purpose of statistical control) and cognitive response to narrative messages was not the focus of our study’s rationale or design, a few noteworthy patterns emerged in the multivariable models presented in S2 Table and S3 Table. Democrats were more likely than Republicans to empathize with the main character (*p* <. 001), and both Democrats (*p* <. 001) and Independents (*p* = .001) were more likely than Republicans to support obesity prevention policies. Women were more likely than men to see themselves as similar to the main character (*p* = .01), empathize with her (*p* = .004), and support obesity prevention policies (*p* <. 001). Finally, obese respondents were more likely than normal weight respondents to counterargue the narratives (*p* <. 001), and both obese and overweight respondents (*p*s <. 001) felt more similar to the character than normal weight respondents.

Turning to the possibility that effects of obesity narratives may differ by demographic factors, we tested RQ1 with interaction terms between political party (Republicans as reference group) and randomized condition in predicting obesity policy support. Interactions between political party and condition were not statistically significant (*p*s >. 22). We also conducted a series of exploratory analyses (as suggested by a blind reviewer) to test whether the effects of message conditions on policy support differed by age, sex, race, formal education, or weight status. These analyses included 23 different statistical tests since many of these variables had multiple categories. Of these 23 tests, only 2 had *p*-values less than 0.10, exactly the number that would be expected by chance alone given. We thus conclude that none of these demographic characteristics significantly influenced the impact of the obesity narratives on policy support.

## Discussion

Results suggest that failure to acknowledge individual responsibility in a narrative to increase support for obesity policy can undermine the narrative’s impact, across the political spectrum. Respondents exposed to stories that explicitly acknowledged individual responsibility, while emphasizing social determinants of obesity, had higher levels of support for obesity policies than those exposed to a narrative that did not acknowledge individual responsibility at all. This occurred because those exposed to the high individual responsibility narrative were less likely to counterargue the story and felt more empathy for the story’s protagonist than those exposed to a narrative that did not acknowledge individual responsibility.

### Theoretical implications

These findings highlight the importance of considering both cognitive responses and character perceptions in response to policy narratives. Relatively few studies have examined how plot, setting, and character features shape responses to policy narratives [4, 5]. Theorists argue that narratives may be persuasive because they reduce the tendency to counterargue a message [23]. Our results are consistent with this argument and further emphasize that strategic decisions about narrative design shape whether or not counterarguing occurs. Theorizing about narrative persuasion has also focused attention on the roles of empathy and perceived similarity with story characters [24]. Our findings suggest that failure to generate empathetic feelings for the protagonist may be particularly detrimental in efforts to persuade. Empathetic responses to a story appear to vary considerably by the way a character’s traits and behavior are described.

### Practical implications

Practically speaking, study results (along with Gollust and Cappella [13]) suggest that communicators should consider some degree of acknowledgement of individual responsibility in efforts to promote support for public policies to address health issues like obesity. While one previous study suggested that failure to acknowledge individual responsibility offered a persuasive advantage among conservatives only, albeit without any theoretical explanation, we failed to replicate this finding. [8] The current study’s results were consistent across political groups and were accompanied by clear theoretical mechanisms to explain the findings. The consistency and theoretical explanation of these findings, combined with the use of a nationally representative sample, lends credence to the reliability and generalizability of these results.

None of the narratives tested here were successful at increasing support for obesity policies relative to a no-exposure control group. While the current study makes clear that a failure to acknowledge individual responsibility at all is likely to be detrimental, the degree of emphasis to place on individual and societal responsibility in strategic narratives to promote health-related policy remains a difficult challenge. Previous work offers some clues, however.

A previous study tested three degrees of acknowledging individual responsibility (high, moderate, and none) and found that the moderate acknowledgement condition was also successful at increasing policy support [8]. This finding was accompanied by support for a proposed theoretical mechanism: increasing the degree to which respondents integrated individual and societal-level determinants of obesity (complex integration). While similar cautions apply to the study sample, the latter finding is on more stable ground due to the presence of a plausible theoretical explanation.

Obesity results from complex interactions between individual decisions and the environment in which those decisions are made. [2] Individuals make decisions every day about what to eat and how active to be, but these decisions are shaped by the social, environmental, economic and physical environment in which they are made. Too much emphasis in strategic messages on individual agency may undermine support for policy interventions, [2] but too little emphasis may also undermine their effectiveness. Messages that underscore the complex relationship between individual decisions and their social context may hold substantial potential for shifting the public toward greater support for policy solutions that target social determinants of obesity. [8] Future research should continue to develop and test messages that successfully strike a balance between acknowledging individual decisions and emphasizing social causes of health issues. In the meantime, results underscore the need to pre-test messages to ensure that they invite cognitions (complex integration over counter-arguments) and character perceptions (affective empathy) that invite favorable responses to an obesity-related narrative.

### Study limitations and future directions

Some of these findings differ from previous (similar) studies, highlighting the importance of further research on effects and mechanisms of narratives to influence health-related policy support. This study’s use of the GfK panel was intended to address limitations of convenience samples utilized in previous work, but the GfK panel over-represents some groups relative to US Census estimates. Nevertheless, GfK’s use of probability-based sampling and use of statistical controls for demographic characteristics in all tests of study hypotheses reduce these concerns.

The current study focused on one type of persuasive message (a strategic narrative designed to promote health policy) with a single character (a woman) in a single context (obesity). It is not known whether lessons learned in the current study can generalize to other forms of strategic messaging around obesity or other topics, or whether responses to the narrative might have differed if the story had focused on a male protagonist. Future work should test whether audiences respond differently to characters of different sex, age, race, and/or educational background. Future research should examine the extent to which variations in the degree to which message acknowledge individual responsibility shapes their impact on policy support in other forms of persuasive communication, including public service announcements, news articles, and policy briefs. Future work should also explore the generalizability of these findings to other health domains like tobacco use, alcohol use, and drug abuse, where public discourse and news coverage often frame debates around individual versus societal responsibility. [25]

## Conclusions

Obesity results from complex interactions between individual decisions and the environment in which people make those decisions [2]. Failure to acknowledge individual responsibility in narratives emphasizing social determinants of obesity has potential to undermine the persuasiveness of policy narratives. Omitting information about individual responsibility, a strongly-held American value, invites the public to counterargue the narratives and reduces feelings of empathy for a character that experiences the challenges and benefits of social determinants of obesity. Such public reactance, in turn, undermines support for policies to reduce rates of obesity. Future research should develop and test narratives that fruitfully emphasize societal causes of other health and social issues amenable to policy intervention.

## Supporting Information

S1 Table Demographics of Obesity Narrative Attributions Study Participants, United States Adults [N = 718], 2011. Abbreviation: N/A = not applicable; N/R = not reported; *n* = sample size; BMI = body mass index; *M* = mean; *SD* = standard deviation. *Note*. For US Demographic Composition, comparison data were extracted from the March 2013 Current Population Survey. For political party affiliation, comparison data are from the 2012 American National Election Study (NES). For weight status, comparison data come from the 2011–2012 National Health and Nutrition Examination Survey. The chi-square test for BMI excluded underweight respondents because their inclusion violated the 5 observations per cell requirement. The demographic information presented in the second column of this table appeared in a paper that was published previously in Preventing Chronic Disease (http://www.cdc.gov/pcd/issues/2013/13_0163.htm). That paper examined different outcomes (intentions to engage in diet and exercise). All other data presented are original to this paper.
(DOCX)

S2 Table Logistic Regression Models Examining Predictors of Simple Elaboration, Counterelaboration, and Counterarguing, United States, 2011. Abbreviation: H = hypothesis; N = sample size; BMI = body mass index. *Note*. All models also included controls for US Census region, metropolitan area, and Internet access.
(DOCX)

S3 Table OLS Regression Models Examining Predictors of Perceived Similarity, Affective Empathy, and Support for Policies to Reduce Obesity, United States, 2011. Abbreviation: OLS = ordinary least squares; H = hypothesis; N = sample size; BMI = body mass index. *Note*. All models also included controls for US Census region, metropolitan area, and Internet access. ^a^Denotes significant difference from the no individual responsibility condition, p = 0.036
(DOCX)

S4 Table Establishing Mediation: OLS Regressions Predicting Support for Public Policies to Reduce Obesity, United States, 2011. Abbreviation: OLS = ordinary least squares; H = hypothesis; N = sample size. *Note*. All models also included controls for political party identification, age, gender, race, education, Body Mass Index (BMI), US Census region, metropolitan area, and Internet access. H6 predicted that a story acknowledging individual responsibility (and emphasizing social causes) would produce more support for policies to reduce rates of obesity than a story that does not acknowledge individual responsibility (H6). We further suggested that this relationship will be mediated by reduced counterarguing (H7), increased perceived similarity (H8), and increased empathy (H9).
(DOCX)

S1 Dataset (DTA)

S1 Syntax (DOC)

S1 Survey Codebook (DOC)

S1 Thought Codebook (DOC)
