# Supplementary material for: Acknowledging Individual Responsibility while Emphasizing Social Determinants in Narratives to Promote Obesity-Reducing Public Policy: A Randomized Experiment
Source: PLoS One. 2015 Feb 23;10(2):e0117565. doi: 10.1371/journal.pone.0117565 (PMC4338108; doi:10.1371/journal.pone.0117565)
Supplement: S1 Table — Abbreviation: N/A = not applicable; N/R = not reported; n = sample size; BMI = body mass index; M = mean; SD = standard deviation. Note. For US Demographic Composition, comparison data were extracted from the March 2013 Current Population Survey. For political party affiliation, comparison data are from the 2012 American National Election Study (NES). For weight status, comparison data come from the 2011–2012 National Health and Nutrition Examination Survey. The chi-square test for BMI excluded underweight respondents because their inclusion violated the 5 observations per cell requirement. The demographic information presented in the second column of this table appeared in a paper that was published previously in Preventing Chronic Disease (http://www.cdc.gov/pcd/issues/2013/13_0163.htm). That paper examined different outcomes (intentions to engage in diet and exercise). All other data presented are original to this paper. (DOCX) [file pone.0117565.s001.docx]

|  | US Demographic Composition | Analytic Sample Proportion (n) or *M* (*SD*) | Test for Differences by Randomized Condition χ^2^/p-value; F/p-value |
| --- | --- | --- | --- |
| **Randomized Condition** |  |  |  |
| No Exposure Control Group | N/A | 0.10 (75) |  |
| No Individual Responsibility | N/A | 0.46 (333) |  |
| High Individual Responsibility | N/A | 0.43 (310) |  |
| **Political Party** |  |  | χ^2^(6) = 7.99, *p* = 0.24 |
| Republican | 0.25 | 0.28 (198) |  |
| Democrat | 0.41 | 0.37 (264) |  |
| Independent | 0.33 | 0.29 (205) |  |
| Something Else | 0.01 | 0.07 (50) |  |
| **Age (mean)** | N/R | 48.26 (17.02) | F(2,715) = 0.74, *p* = 0.48 |
| **Female sex** | 0.52 | 0.53 (380) | χ^2^(2) = 3.20, *p* = 0.20 |
| **Race/Ethnicity** |  |  | χ^2^(8) = 7.34, *p* = 0.50 |
| White, Non-Hispanic | 0.72 | 0.74 (533) |  |
| Black, Non-Hispanic | 0.10 | 0.09 (64) |  |
| Other, Non-Hispanic | 0.07 | 0.04 (26) |  |
| Hispanic | 0.11 | 0.09 (66) |  |
| 2+ Races, Non-Hispanic | N/R | 0.04 (29) |  |
| **Highest Level of Education Completed** |  |  | χ^2^(6) = 4.24, *p* = 0.65 |
| Less than High School Diploma | 0.11 | 0.08 (56) |  |
| High School Diploma or Equivalent | 0.30 | 0.29 (206) |  |
| Some College | 0.30 | 0.30 (217) |  |
| Bachelor’s Degree or Higher | 0.30 | 0.33 (239) |  |
| **Weight Status in Body Mass Index (BMI)** |  |  | χ^2^(4) = 6.10, *p* = 0.19 |
| Underweight (BMI <18.5) | N/R | 0.01 (10) |  |
| Normal Weight (BMI >=18.5 and <25) | N/R | 0.39 (272) |  |
| Overweight (BMI >=25 and <30) | 0.34 | 0.30 (211) |  |
| Obese (BMI >=30) | 0.35 | 0.30 (210) |  |
