# Supplementary material for: Acknowledging Individual Responsibility while Emphasizing Social Determinants in Narratives to Promote Obesity-Reducing Public Policy: A Randomized Experiment
Source: PLoS One. 2015 Feb 23;10(2):e0117565. doi: 10.1371/journal.pone.0117565 (PMC4338108; doi:10.1371/journal.pone.0117565)
Supplement: S2 Table — Abbreviation: H = hypothesis; N = sample size; BMI = body mass index. Note. All models also included controls for US Census region, metropolitan area, and Internet access. (DOCX) [file pone.0117565.s002.docx]

|  | Simple Elaboration (H1) | Counterelaboration (H2) | Counterarguing (H3) |
| --- | --- | --- | --- |
|  | Coefficient (p) | Coefficient (p) | Coefficient (p) |
| **Randomized Condition** |  |  |  |
| No Exposure Control |  |  |  |
| No Individual Responsibility | reference | reference | Reference |
| High Individual Responsibility | 0.02 (0.91) | 0.61 (<0.001) | –0.81 (<0.001) |
| **Political Party** |  |  |  |
| Republican | reference | reference | Reference |
| Democrat | 0.41 (0.07) | 0.15 (0.50) | –0.31 (0.25) |
| Independent | 0.04 (0.87) | 0.08 (0.73) | –0.05 (0.87) |
| Something Else | 0.23 (0.52) | 0.71 (0.06) | –0.24 (0.61) |
| **Age (centered)** | 0.00 (0.85) | 0.01 (0.14) | 0.01 (0.30) |
| **Female sex (vs. male)** | 0.05 (0.77) | 0.30 (0.08) | –0.29 (0.18) |
| **Race/Ethnicity** |  |  |  |
| White, Non-Hispanic | reference | reference | Reference |
| Black, Non-Hispanic | –0.38 (0.22) | 0.01 (0.97) | –1.10 (0.03) |
| Other, Non-Hispanic | –2.55 (<0.001) | 0.04 (0.93) | –0.57 (0.35) |
| Hispanic | –0.44 (0.18) | –0.41 (0.22) | –0.37 (0.44) |
| 2+, Non-Hispanic | –0.31 (0.48) | 0.15 (0.75) | 0.26 (0.61) |
| **Level of Education** |  |  |  |
| Less than High School Diploma | reference | reference | Reference |
| High School Diploma | 0.46 (0.19) | 0.02 (0.96) | –0.29 (0.54) |
| Some College | 0.36 (0.31) | 0.30 (0.39) | –0.15 (0.75) |
| Bachelor’s Degree or More | 0.61 (0.08) | 0.27 (0.44) | 0.23 (0.61) |
| **Body Mass Index (BMI)** |  |  |  |
| Normal (BMI >=18.5 & <25) | reference | reference | Reference |
| Underweight (BMI <18.5) | –0.17 (0.88) | 0.17 (0.83) | –0.27 (0.75) |
| Overweight (BMI >=25 & <30) | 0.16 (0.12) | –0.16 (0.43) | –0.39 (0.11) |
| Obese (BMI >=30) | 0.15 (0.08) | –0.15 (0.48) | –1.10 (<.001) |
| Constant | –1.08 (0.10) | –0.64 (0.28) | –1.41 (0.07) |
| **Model Statistics** |  |  |  |
| Pseudo R-Squared | 0.06 | 0.04 | 0.10 |
| N | 629 | 629 | 629 |
