# Supplementary material for: Acknowledging Individual Responsibility while Emphasizing Social Determinants in Narratives to Promote Obesity-Reducing Public Policy: A Randomized Experiment
Source: PLoS One. 2015 Feb 23;10(2):e0117565. doi: 10.1371/journal.pone.0117565 (PMC4338108; doi:10.1371/journal.pone.0117565)
Supplement: S3 Table — Abbreviation: OLS = ordinary least squares; H = hypothesis; N = sample size; BMI = body mass index. Note. All models also included controls for US Census region, metropolitan area, and Internet access. aDenotes significant difference from the no individual responsibility condition, p = 0.036 (DOCX) [file pone.0117565.s003.docx]

|  | Perceived Similarity (H4) | Affective Empathy (H5) | Obesity Policy Support (H6) |
| --- | --- | --- | --- |
|  | Coefficient (p) | Coefficient (p) | Coefficient (p) |
| **Randomized Condition** |  |  |  |
| No Exposure Control |  |  | Reference |
| No Individual Responsibility | Reference | reference | –0.15 (0.11) |
| High Individual Responsibility | 0.55 (<.001) | 0.30 (<0.001) | –0.03 (0.76)^a^ |
| **Political Party** |  |  |  |
| Republican | Reference | reference | Reference |
| Democrat | 0.12 (0.19) | 0.29 (<0.001) | 0.65 (<0.001) |
| Independent | 0.03 (0.75) | 0.10 (0.20) | 0.24 (0.001) |
| Something Else | –0.16 (0.27) | –0.06 (0.59) | 0.05 (0.65) |
| **Age (centered)** | 0.00 (0.66) | 0.004 (0.02) | –0.01 (0.001) |
| **Female sex (vs. male)** | 0.21 (0.01) | 0.16 (0.004) | 0.20 (<0.001) |
| **Race/Ethnicity** |  |  |  |
| White, Non-Hispanic | Reference | reference | Reference |
| Black, Non-Hispanic | 0.13 (0.33) | 0.13 (0.21) | 0.10 (0.30) |
| Other, Non-Hispanic | 0.25 (0.19) | 0.08 (0.61) | 0.03 (0.83) |
| Hispanic | 0.13 (0.33) | –0.15 (0.18) | 0.11 (0.28) |
| 2+, Non-Hispanic | 0.14 (0.43) | –0.08 (0.57) | 0.03 (0.81) |
| **Level of Education** |  |  |  |
| Less than High School Diploma | Reference | reference | Reference |
| High School Diploma | –0.07 (0.64) | –0.17 (0.14) | –0.22 (0.06) |
| Some College | –0.17 (0.24) | –0.25 (0.04) | –0.15 (0.20) |
| Bachelor’s Degree or More | –0.31 (0.04) | –0.36 (0.002) | –0.14 (0.22) |
| Body Mass Index (BMI) |  |  |  |
| Normal (BMI >=18.5 & <25) | Reference | reference | Reference |
| Underweight (BMI <18.5) | 0.10 (0.76) | 0.45 (0.09) | 0.37 (0.11) |
| Overweight (BMI >=25 & <30) | 0.37 (<0.001) | 0.01 (0.86) | 0.04 (0.51) |
| Obese (BMI >=30) | 0.60 (<0.001) | 0.13 (0.06) | 0.00 (0.99) |
| Constant | 2.13 (<0.001) | 2.47 (<0.001) | 3.38 (<0.001) |
| **Model Statistics** |  |  |  |
| Model R-Squared | 0.22 | 0.16 | 0.21 |
| N | 608 | 618 | 680 |
