# Supplementary material for: Acknowledging Individual Responsibility while Emphasizing Social Determinants in Narratives to Promote Obesity-Reducing Public Policy: A Randomized Experiment
Source: PLoS One. 2015 Feb 23;10(2):e0117565. doi: 10.1371/journal.pone.0117565 (PMC4338108; doi:10.1371/journal.pone.0117565)
Supplement: S4 Table — Abbreviation: OLS = ordinary least squares; H = hypothesis; N = sample size. Note. All models also included controls for political party identification, age, gender, race, education, Body Mass Index (BMI), US Census region, metropolitan area, and Internet access. H6 predicted that a story acknowledging individual responsibility (and emphasizing social causes) would produce more support for policies to reduce rates of obesity than a story that does not acknowledge individual responsibility (H6). We further suggested that this relationship will be mediated by reduced counterarguing (H7), increased perceived similarity (H8), and increased empathy (H9). (DOCX) [file pone.0117565.s004.docx]

|  | Coefficient (p) (H6) | Coefficient (p) (H7-H9) | Coefficient (p) (H7-H9) |
| --- | --- | --- | --- |
| **Randomized Condition** |  |  |  |
| No Individual Responsibility | Reference |  | Reference |
| High Individual Responsibility | 0.13 (0.03) |  | 0.01 (0.93) |
| **Cognitive Responses and Character Perceptions** |  |  |  |
| Counterelaboration |  | 0.09 (0.10) | 0.09 (0.11) |
| Counterarguing |  | –0.21 (0.003) | –0.21 (0.003) |
| Perceived Similarity |  | –0.02 (0.60) | –0.02 (0.59) |
| Affective Empathy |  | 0.31 (<0.001) | 0.31 (<0.001) |
| **Model Statistics** |  |  |  |
| Model R-Squared | 0.22 | 0.32 | 0.32 |
| N | 581 | 581 | 581 |
