# Supplementary material for: Acknowledging Individual Responsibility while Emphasizing Social Determinants in Narratives to Promote Obesity-Reducing Public Policy: A Randomized Experiment
Source: PLoS One. 2015 Feb 23;10(2):e0117565. doi: 10.1371/journal.pone.0117565 (PMC4338108; doi:10.1371/journal.pone.0117565)
Supplement: S1 Syntax — (DOC) [file pone.0117565.s006.doc]

**STATA Syntax for Survey Data Recodes and Analysis**

Table 1 Recodes and Analyses

*Creating individual responsibility condition variable (3 categories)

gen condr = DOV_Message

recode condr (9=1) (1=2) (2=2) (3=2) (4=2) (5=3) (6=3) (7=3) (8=3)

*Creating individual responsibility condition variable without the control group (2 categories)

gen condr2 = condr

recode condr2 (1=.)

*Creating 4-category political party variable

gen polparty = Q23_1

recode polparty (-1=.)

*Creating age variable

Gen age = PPAGE

*Creating sex variable

gen sex = PPGENDER

recode sex (1=0) (2=1)

*Creating race/ethnicity variable

gen race = PPETHM

*Creating 4-category education variable

gen educ = PPEDUCAT

order

*Creating continuous and 4-category BMI variables

gen inches2 = Q25_Inches

recode inches2 (-1=.)

gen inches1 = Q25_Feet

recode inches1 (-1=.) (2=.) (4=48) (5=60) (7=72)

gen height = inches1+inches2

gen pounds = Q26

recode pounds (-1=.) (50=.)

gen bmi = pounds/(height*height*703)

gen bmi4 = bmi

replace bmi4 = 1 if bmi<18.5

replace bmi4 = 2 if (bmi>=18.5 & bmi<25)

replace bmi4 = 3 if (bmi>=25 & bmi<30)

replace bmi4 = 4 if (bmi>=30 & bmi<99)

gen bmi4a = bmi4

recode bmi4a (1=2) (2=1)

*Running all analyses presented in Table 1

tab condr

tab polparty

sum age

tab sex

tab race

tab educ

tab bmi4

tab polparty condr, chi

anova age condr

tab sex condr, chi

tab race condr, chi

tab educ condr, chi

tab bmi4 condr, chi

*(re-running BMI chi-square excluding underweight which violates 5 observations per-cell requirements for chi-square)

gen bmi4b = bmi4

replace bmi4b = . if bmi4==1

tab bmi4b condr, chi

Dependent Variable and Manipulation Check Variable Recodes and Scale-Building

*Creating perceived individual and societal responsibility manipulation check variables

gen percIRa = Q2_A

recode percIRa (-1=.)

gen percIRb = Q2_C

recode percIRb (-1=.)

gen percIRc = Q2_E

recode percIRc (-1=.)

alpha percIRa percIRb percIRc

gen percIR = (percIRa+percIRb+percIRc)/3

gen percSRa = Q2_B

recode percSRa (-1=.)

gen percSRb = Q2_D

recode percSRb (-1=.)

gen percSRc = Q2_F

recode percSRc (-1=.)

alpha percSRa percSRb percSRc, item

corr percSRa percSRb percSRc

gen percSR = (percSRa+percSRb)/2

*Manipulation check for responsibility manipulation

ttest percIR, by(condr2)

ttest percIR = 3 if condr2==2

ttest percIR = 3 if condr2==3

ttest percSR, by(condr2)

ttest percSR = 3 if condr2==2

ttest percSR = 3 if condr2==3

*Creating perceived similarity, affective empathy, and policy support variables

alpha Q12_A Q12_B Q12_C Q12_D Q12_E Q12_F

gen similar = (Q12_A + Q12_B + Q12_C + Q12_D + Q12_E + Q12_F)/6

sum similar

alpha Q10_A Q10_B Q10_C Q4_C Q4_D

gen aempathy = (Q10_A +Q10_B +Q10_C +Q4_C +Q4_D)/5

sum aempathy

alpha Q5_A Q5_B Q5_C Q5_D Q5_E Q5_F Q5_G Q5_H

gen support = (Q5_A +Q5_B +Q5_C +Q5_D +Q5_E +Q5_F +Q5_G +Q5_H)/8

sum support

*Creating empathy condition variable (2 categories, excluding control group; 1 = empathy, 2 = not)

gen conde = DOV_Message

recode conde (9=.) (1=1) (2=2) (3=1) (4=2) (5=1) (6=2) (7=1) (8=2)

*Creating partisan cue condition variable (2 categories, excluding control group; 1 = Dem, 2 = Rep)

gen condp = DOV_Message

recode condp (9=.) (1=1) (2=1) (3=2) (4=2) (5=1) (6=1) (7=2) (8=2)

*Manipulation check for empathy manipulation

ttest aempathy, by(conde)

*Manipulation check for partisan cue similarity manipulation

ttest similar if polparty==1, by(condp)

*Tabulation of various thought categories

tab celab

tab selab

tab cargue

tab complex

Table 2 Analyses

*centering age for all analyses

gen age2 = age- 48.25905

*Running all analyses presented in Table 2

xi: logit selab i.condr2 i.polparty age2 sex i.race i.educ i.bmi4a i.ppreg9 i.PPMSACAT i.PPNET

xi: logit celab i.condr2 i.polparty age2 sex i.race i.educ i.bmi4a i.ppreg9 i.PPMSACAT i.PPNET

xi: logit cargue i.condr2 i.polparty age2 sex i.race i.educ i.bmi4a i.ppreg9 i.PPMSACAT i.PPNET

Table 3 Analyses

*Running all analyses presented in Table 3

xi: reg similar i.condr2 i.polparty age2 sex i.race i.educ i.bmi4a i.ppreg9 i.PPMSACAT i.PPNET, beta

xi: reg aempathy i.condr2 i.polparty age2 sex i.race i.educ i.bmi4a i.ppreg9 i.PPMSACAT i.PPNET, beta

xi: reg support i.condr i.polparty age2 sex i.race i.educ i.bmi4a i.ppreg9 i.PPMSACAT i.PPNET, beta

test _Icondr_2 = _Icondr_3

Table 4 Analyses

*Running all analyses presented in Table 4

xi: reg support i.condr2 i.polparty age2 sex i.race i.educ i.bmi4a i.ppreg9 i.PPMSACAT i.PPNET if similar > .9 & similar < 5.1 & aempathy > .9 & aempathy < 5.1, beta

xi: reg support celab cargue similar aempathy i.polparty age2 sex i.race i.educ i.bmi4a i.ppreg9 i.PPMSACAT i.PPNET, beta

xi: reg support i.condr2 celab cargue similar aempathy i.polparty age2 sex i.race i.educ i.bmi4a i.ppreg9 i.PPMSACAT i.PPNET, beta

Formal Test of Mediation

Syntax for Mediation analysis in SPSS (requires the PROCESS macro written by Andrew F. Hayes, The Ohio State University, [hayes.338@osu.edu](mailto:hayes.338@osu.edu) and publicly available at <http://www.afhayes.com/spss-sas-and-mplus-macros-and-code.html>)

INDIRECT y = support / x = condr2 / m = celab cargue similar aempathy indep dem oth age2 gender black othrac hisp tworac hsed somec coll under over obese reg2 reg3 reg4 reg5 reg6 reg7 reg8 reg9 PPMSACAT PPNET / c = 25 / boot = 5000 / conf = 95 / normal = 1 / contrast = 1 / percent = 1 / bc = 1 / bca = 0.

Testing for Interactions

*Interactions by Political Party (RQ1)

xi: reg support i.condr*i.polparty age2 sex i.race i.educ i.bmi4a i.ppreg9 i.PPMSACAT i.PPNET

*Interactions by Other Demographic Variables

xi: reg support i.condr*sex i.polparty age2 i.race i.educ i.bmi4a i.ppreg9 i.PPMSACAT i.PPNET

xi: reg support i.condr*i.race i.polparty age2 sex i.educ i.bmi4a i.ppreg9 i.PPMSACAT i.PPNET

xi: reg support i.condr*i.educ i.polparty age2 sex i.race i.bmi4a i.ppreg9 i.PPMSACAT i.PPNET

xi: reg support i.condr*i.bmi4a i.polparty age2 sex i.race i.educ i.ppreg9 i.PPMSACAT i.PPNET

xi: reg support i.condr*age2 i.polparty sex i.race i.educ i.bmi4a i.ppreg9 i.PPMSACAT i.PPNET
