# Supplementary material for: Acknowledging Individual Responsibility while Emphasizing Social Determinants in Narratives to Promote Obesity-Reducing Public Policy: A Randomized Experiment
Source: PLoS One. 2015 Feb 23;10(2):e0117565. doi: 10.1371/journal.pone.0117565 (PMC4338108; doi:10.1371/journal.pone.0117565)
Supplement: S1 Survey Codebook — (DOC) [file pone.0117565.s007.doc]

Attributions of Responsibility for Obesity

July 2011

- Study Details -

Note: This page may be removed when the questionnaire is sent to the client. However, it must exist in the version sent to Operations.

| **Survey Name** | **Attributions of Responsibility for Obesity** |
| --- | --- |
| **Client Name** | **Cornell University** |

NOTE: Variable names are highlighted in **YELLOW** throughout this codebook.

**AUTO-RECORDED VARIABLES:**

**CaseID** = unique individual identifier code (number; non-identifiable)

**weight** = survey weights calculated by Knowledge Networks (not used in the study)

**duration** = interview duration in minutes

**Study Design & Documentation**

**Introduction & Sample Definition**

In July and August of 2011 Knowledge Networks conducted the Attributions of Responsibility for Obesity Survey on behalf of Cornell University. A national general population sample age 18+ was selected from KnowledgePanel, a probability-based web panel designed to be representative of the United States. The survey consists of two stages: consent to participate in the survey and the main survey with the consented respondents. A pretest survey was conducted to verify the survey functionality and duration.

**Survey Completion and Sample Sizes**

The number of respondents sampled and who participated in the survey for the main interview are presented below. The completion goal for the study was to collect at least 600 completes.

**Key Survey Response Statistics: Main Interview**

| **Field** | **Field** | **Sampled** | **Completed** | **Completed** | **Consented** | **Consent** |
| --- | --- | --- | --- | --- | --- | --- |
| **Period Start** | **Period End** |  |  | **Rate** |  | **Rate** |

7/28/11 8/11/2011 1,462 904 62% 718 79%

**Knowledge Networks Methodology**

**Introduction**

Knowledge Networks (KN) has recruited the first online research panel that is representative of the entire U.S. population. Panel members are randomly recruited through probability-based sampling, and households are provided with access to the Internet and hardware if needed.

Knowledge Networks selects households by using address-based sampling methods; formerly, KN relied on random-digit dialing (RDD) Once households are recruited for the panel, they are contacted by e-mail for survey taking or panelists visit their online member page for survey taking (instead of being contacted by phone or postal mail). This allows surveys to be fielded very quickly and economically. In addition, this approach reduces the burden placed on respondents, since e-mail notification is less intrusive than telephone calls, and most respondents find answering Web questionnaires more interesting and engaging than being questioned by a telephone interviewer. Furthermore, respondents have the freedom to choose what time of day to participate in research.

Documentation regarding KnowledgePanel sampling, data collection procedures, weighting, and

IRB-bearing issues are available at the below online resources.

<http://www.knowledgenetworks.com/ganp/reviewer-info.html>[h](http://www.knowledgenetworks.com/knpanel/index.html)ttp://www.knowledgenetworks.com/knpanel/index.html [h](http://www.knowledgenetworks.com/ganp/irbsupport/)ttp://www.knowledgenetworks.com/ganp/irbsupport/

**Panel Recruitment Methodology**

When Knowledge Networks began recruiting in 1999, the company established the first online research panel (now called KnowledgePanel®) based on probability sampling covering both the online and offline populations in the U.S. Panel members are recruited through national random samples, originally by telephone and now almost entirely by postal mail. Households are provided with access to the Internet and hardware if needed. Unlike Internet convenience panels, also known as “opt-in” panels, that includes only individuals with Internet access who volunteer themselves for research, KnowledgePanel recruitment uses dual sampling frames that includes both listed and unlisted telephone numbers, telephone and non-telephone households, and cell- phone-only households, as well as households with and without Internet access. Only persons sampled through these probability-based techniques are eligible to participate on KnowledgePanel. Unless invited to do so as part of these national samples, no one on their own can volunteer to be on the panel.

**RDD and ABS Sample Frames**

KnowledgePanel members today could have been recruited by either the former random digit dialing (RDD) sampling or the current address-based sampling (ABS) methodologies In this section, we will describe the RDD-based methodology; the ABS methodology is described in a separate section below. To offset attrition, multiple recruitment samples are fielded evenly throughout the calendar year.

KnowledgePanel recruitment methodology has used the quality standards established by selected RDD surveys conducted for the Federal government (such as the CDC-sponsored National Immunization Survey).

KN employed list-assisted RDD sampling techniques based on a sample frame of the U.S. residential landline telephone universe. For purposes of efficiency, KN excludes only those banks of telephone numbers (a bank consists of 100 numbers) that had fewer than two directory listings. Additionally, an oversampling was conducted within a stratum of telephone exchanges that had high concentrations of African American and Hispanic households based on Census data. Note that recruitment sampling is done without replacement, thus numbers already fielded do not get fielded again.

A telephone number for which a valid postal address can be matched occurred in about 67-70% of each sample. These address-matched cases were all mailed an advance letter informing them that they had been selected to participate in KnowledgePanel. For purposes of efficiency, the unmatched numbers were most recently under-sampled at a rate of 0.75 relative to the matched numbers. Both the minority oversampling mentioned above and this under-sampling of non- address households are adjusted appropriately in the panel’s weighting procedures.

Following the mailings, telephone recruitment by trained interviewers/recruiters begins for all sampled telephone numbers. Telephone numbers for cases sent to recruiters were dialed for up to 90 days, with at least 14 dial attempts for cases in which no one answers the phone, and for numbers known to be associated with households. Extensive refusal conversion was also performed. The recruitment interview, about 10 minutes in length, begins with informing the household member that the household had been selected to join KnowledgePanel. If the household does not have a computer and access to the Internet, the household member is told that in return for completing a short survey weekly, the household will be provided with free monthly Internet access and a laptop computer (in the past, the household was provided with a WebTV device). All members of the household are enumerated, and some initial demographic and background information on prior computer and Internet use was collected.

Households that informed recruiters that they had a home computer and Internet access were asked to take KN surveys using their own equipment and Internet connection. Incentive points per survey, redeemable for cash, are given to these “PC” (personal computer) respondents for completing their surveys. Panel members provided with a laptop computer and free Internet access do not participate in this per-survey points-incentive program. However, all panel members do receive special incentive points for select surveys to improve response rates and for all longer surveys as a modest compensation for the extra burden of their time and participation.

For those panel members receiving a laptop computer, each unit is custom-configured prior to shipment with individual email accounts so that it is ready for immediate use by the household. Most households are able to install the hardware without additional assistance, although KN maintains a toll-free telephone line for technical support. The KN Call Center contacts household members who do not respond to e-mail and attempts to restore both contact and participation.

PC panel members provide their own e-mail addresses, and we send their weekly survey invitations to that e-mail account.

All new panel members receive an initial survey for the dual purpose of welcoming them as new panel members and introducing them to how online survey questionnaires work. New panel members also complete a separate profile survey that collects essential demographic information such as gender, age, race, income, and education to create a personal member profile. This information can be used to determine eligibility for specific studies and is factored in for weighting purposes. Operationally, once the profile information is stored, it does not need to be re-collected as a part of each and every survey. This information is also updated annually for all panel members. Once new members have completed their profile surveys, they are designated as “active,” and considered ready to be sampled for client studies. [Note: Parental or legal guardian consent is also collected for the purpose of conducting surveys with teenage panel members,

aged 13 to17.]

Once a household is recruited and each household member’s e-mail address is either obtained or provided, panel members are sent survey invitations linked through a personalized e-mail message (instead of by phone or postal mail). This contact method permits surveys to be fielded quickly and economically, and also facilitates longitudinal research. In addition, this approach reduces the burden placed on respondents, since e-mail notification is less intrusive than telephone calls and allows research subjects to participate in research when it is convenient for them.

**Address-Based Sampling (ABS) Methodology**

When KN first started panel recruitment in 1999, the conventional opinion among survey experts was that probability-based sampling could be carried out cost effectively through the use of a national RDD samples. The RDD landline frame at the time allowed access to 96% of U.S. households. This is no longer the case. In 2009, Knowledge Networks introduced use of the ABS sample frame to panel recruitment to reflect the real changes in society and telephony over recent years. Those changes that have reduced the long-term scientific viability of landline RDD sampling methodology are as follows: declining respondent cooperation in telephone surveys as reflected in “do not call” lists, call screening, caller-ID devices, and answering machines; dilution of the RDD sample frame as measured by the working telephone number rate; and finally, the emergence of cell phone-only households (CPOHH) because such households are excluded from the RDD frame because they have no landline telephone.

According to the Centers for Disease Control and Prevention (January-June 2010), approximately 28.6% of all U.S. households cannot be contacted through RDD sampling—26.6% as a result of CPOHH status and 2% because they have no telephone service whatsoever. Among some age segments, the RDD non-coverage would be substantial: 40% of young adults, ages 18–24, reside in CPOHHs, 51% of those ages 25–29, and 40% of those ages 30–34 (see Blumberg SJ, Luke JV. Wireless substitution: Early release of estimates from the National Health Interview Survey, January–June 2010. National Center for Health Statistics. December 2010. Available from: [http://www.cdc.gov/nchs/nhis.htm.](http://www.cdc.gov/nchs/nhis.htm))

After conducting an extensive pilot project in 2008, KN made the decision to move toward address-based sample (ABS) frame in response to the growing number of cell-phone- only households that are outside the RDD frame. Before conducting the ABS pilot, we also experimented with supplementing its RDD samples with cell-phone samples. However, this approach would was not cost effective—and raised a number of other operational, data quality, and liability issues (for example, calling cell phones while respondents were driving).

The key advantage of the ABS sample frame is that it allows sampling of almost all U.S. households. An estimated 97% of households is “covered” in sampling nomenclature. Regardless of household telephone status, those households can be reached and contacted through postal mail. Second, the KNABS pilot project revealed several additional advantages beyond expected improvement in recruiting adults from CPOHHs:

Improved sample representativeness for minority racial and ethnic groups improved inclusion of lower educated and low income households Exclusive inclusion of the fraction of CPOHHs that have neither a landline telephone nor Internet access (approximately four to six percent of US households).

ABS involves probability-based sampling of addresses from the U.S. Postal Service’s Delivery Sequence File. Randomly sampled addresses are invited to join KnowledgePanel through a series of mailings and, in some cases, telephone follow-up calls to non-responders when a telephone number can be matched to the sampled address. Operationally, invited households have the option to join the panel by one of several ways:

Completing and returning a paper form in a postage-paid envelope, Calling a toll-free hotline maintained by Knowledge Networks, or

Going to a dedicated KN web site and completing an online recruitment form.

After initially accepting the invitation to join the panel, respondents are then “profiled” online by answering key demographic questions about themselves. This profile is maintained through the same procedures that were previously established for RDD-recruited panel members. Respondents not having an Internet connection are provided a laptop computer and free Internet service. Respondents sampled from the ABS frame, like those sampled from the RDD frame, are offered the same privacy terms and confidentiality protections that we have developed over the years and that have been reviewed by dozens of Institutional Review Boards.

Large-scale ABS sampling for KnowledgePanel recruitment began in April 2009. As a result, sample coverage on KnowledgePanel of CPOHHs, young adults, and non-whites has been increasing steadily since that time.

Because KnowledgePanel members have been recruited from two different sample frames, RDD and ABS, KN implemented several technical processes to merge samples sourced from these frames. KN’s approach preserves the representative structure of the overall panel for the selection of individual client study samples. An advantage of mixing ABS frame panel members in any KnowledgePanel sample is a reduction in the variance of the weights. ABS-sourced samples tend to align more closely to the overall demographic distributions in the population, and thus the associated adjustment weights are somewhat more uniform and less varied. This variance reduction efficaciously attenuates the sample’s design effect and confirms a real advantage for study samples drawn from KnowledgePanel with its dual frame construction.

**Survey Administration**

For client surveys, samples are drawn at random from among active panel members. Depending on the study, eligibility criteria will be applied or in-field screening of the sample will be carried out. Sample sizes can range widely depending on the objectives and design of the study.

Once assigned to a survey, members receive a notification e-mail letting them know there is a new survey available for them to take. This email notification contains a link that sends them to the survey questionnaire. No login name or password is required. The field period depends on the client’s needs and can range anywhere from a few hours to several weeks.

After three days, automatic email reminders are sent to all non-responding panel members in the sample. If email reminders do not generate a sufficient response, an automated telephone reminder call can be initiated. The usual protocol is to wait at least three to four days after the e- mail reminder before calling. To assist panel members with their survey taking, each individual has a personalized “home page” that lists all the surveys that were assigned to that member and have yet to be completed.

Knowledge Networks also operates an ongoing modest incentive program to encourage participation and create member loyalty. Members can enter special raffles or can be entered into special sweepstakes with both cash rewards and other prizes to be won.

The typical survey commitment for panel members is one survey per week or four per month with duration of 10 to 15 minutes per survey. Some client surveys exceed this time, and in the case of longer surveys, an additional incentive can be provided.

**Survey Sampling from KnowledgePanel**

Once Panel Members are recruited and profiled, they become eligible for selection for specific client surveys. In most cases, the specific survey sample represents a simple random sample from the panel, for example, a general population survey. Customized stratified random sampling based on profile data can also be conducted as required by the study design.

The general sampling rule is to assign no more than one survey per week to members. Allowing for rare exceptions during some weeks, this limits a member’s total assignments per month to four or six surveys. In certain cases, a survey sample calls for pre-screening, that is, members are drawn from a subsample of the panel (such as females, Republicans, grocery shoppers, etc.). In such cases, care is taken to ensure that all subsequent survey samples drawn that week are selected in such a way as to result in a sample that remains representative of the panel distributions.

**Sample Weighting**

The design for KnowledgePanel® recruitment begins as an equal probability sample with several enhancements incorporated to improve efficiency. Since any alteration in the selection process is a deviation from a pure equal probability sample design, statistical weighting adjustments are made to the data to offset known selection deviations. These adjustments are incorporated in the sample’s **base weight**.

There are also several sources of survey error that are an inherent part of any survey process,

such as non-coverage and non-response due to panel recruitment methods and to inevitable panel attrition. We address these sources of sampling and non-sampling error by using a **panel demographic post-stratification weight** as an additional adjustment.

All the above weighting is done before the study sample is drawn. Once a study sample is finalized (all data collected and a final data set made), a set of **study-specific post-stratification weights** are constructed so that the study data can be adjusted for the study’s sample design and for survey non-response.

A description of these types of weights follows.

**The Base Weight**

In a KnowledgePanel sample there are seven known sources of deviation from an equal probability of selection design. These are corrected in the Base Weight and are described below.

1. Under-sampling of telephone numbers unmatched to a valid mailing address

An address match is attempted on all the Random Digit Dial (RDD)-generated telephone numbers in the sample after the sample has been purged of business and institutional numbers and screened for non-working numbers. The success rate for address matching is in the 60 to 70% range. Households having telephone numbers with valid addresses are sent an advance letter, notifying them that they will be contacted by phone to join KnowledgePanel. The remaining, unmatched numbers are under-sampled as a recruitment efficiency strategy. Advance letters improve recruitment success rates. Under-sampling was suspended between July 2005 and April 2007. It was resumed in May 2007, using a sampling rate of 0.75. RDD recruitment ended in July 2009.

2. RDD selection proportional to the number of telephone landlines reaching the household

As part of the field data collection operation, information is collected on the number of separate telephone landlines in each selected household. The probability of selecting a multiple-line household is down-weighted by the inverse of the number of landlines. RDD recruitment ended in July 2009.

3. Some minor oversampling of Chicago and Los Angeles in early pilot surveys

Two pilot surveys carried out in Chicago and Los Angeles when the panel was initially being built increased the relative size of the sample from these two cities. With natural attrition and growth in size, that impact is disappearing over time. It remains part of our base adjustment weighting because of a small number of extant panel members from that initial panel cohort.

4. Early oversampling the four largest states and central region states

At the time when the panel was first being built, survey demand in the four largest states (California, New York, Florida, and Texas) necessitated oversampling during January– October 2000. Similarly, the central region states were oversampled for a brief period of time. These now diminishing effects still remain in the panel membership and thus weighting adjustments are required for these geographic areas.

5. Under-sampling of households not covered by the MSN® TV service network

Certain small areas of the U.S. are not serviced by MSN®, thus the MSN®TV units distributed to non-Internet households prior to January 2009 could not be used for those recruited non-Internet households. Overall, the result is a small residual under-sample in those geographic areas which requires a minor weighting adjustment for those locations. Since January 2010, laptop computers with dial-up access are being distributed to non- Internet households thus eliminating this under-coverage component.

6. RDD oversampling of African American and Hispanic telephone exchanges

As of October 2001, oversampling of telephone exchanges with a higher density of minority households (specifically, African American and Hispanic) was implemented to increase panel membership for those groups. These exchanges were oversampled at approximately twice the rate of other exchanges. This oversampling is corrected in the base weight. RDD recruitment ended in July 2009.

7. Address-based sample phone match adjustment

Toward the end of 2008, Knowledge Networks began recruiting panel members by using an address-based sample (ABS) frame in addition to RDD recruitment. Once recruitment through the mail, including follow-up mailings to ABS non-respondents was completed, telephone recruitment was added. Non-responding ABS households where a landline telephone number could be matched to an address were subsequently called and telephone recruitment was initiated. This effort resulted in a slight overall disproportionate number of landline households being recruited in a given ABS sample. A base weight adjustment is applied to return the ABS recruitment panel members to the sample’s correct national proportion of phone-match and no phone-match households.

8. ABS oversample stratification adjustment

In late 2009 the ABS sample began incorporating a geographic stratification design. Census blocks with high density minority communities were oversampled (Stratum 1) and the balance of the census blocks (Stratum 2) were relatively under-sampled. The definition of high density and minority community and the relative proportion between strata differed among specific ABS samples. In 2010, the two strata were redefined to target high density Hispanic areas in Stratum 1 and all else in Stratum 2. In 2011, pre- identified ancillary information and not census block data were used to construct and target four strata as follows: Hispanic ages 18-24, Non-Hispanic ages 18-24, Hispanic ages 25+ and Non-Hispanic ages 25+. An appropriate base weight adjustment is applied to each relevant sample to correct for these stratified designs. Also in 2011, a separate sample targeting only persons ages 18-24 was fielded across the year also using predictive ancillary information. Combined with the four-stratum sample, the base weight adjustment compensates for cases from this unique young adult over-sample.

**The Panel Demographic Post-stratification Weight**

To reduce the effects of any non-response and non-coverage bias in the overall panel membership (before the study sample is drawn), a post-stratification adjustment is applied based on demographic distributions from the most recent data from the Current Population Survey (CPS). The benchmark distributions for Internet access among the U.S. population of adults are obtained from the most recent special CPS supplemental survey measuring Internet access (October 2009).

The overall panel post-stratification variables include:

Gender (Male/Female)

Age (18–29, 30–44, 45–59, and 60+)

Race/Hispanic ethnicity (White/Non-Hispanic, Black/Non-Hispanic, Other/Non- Hispanic, 2+ Races/Non-Hispanic, Hispanic)

Education (Less than High School, High School, Some College, Bachelor and beyond) Census Region (Northeast, Midwest, South, West)

Household income (under $10k, $10K to <$25k, $25K to <$50k, $50K to <$75k, $75K

to <$100k, $100K+)

Home ownership status (Own, Rent/Other) Metropolitan Area (Yes, No)

Internet Access (Yes, No)

The Panel Demographic Post-stratification weight is applied prior to a probability proportional to size (PPS) selection of a study sample from KnowledgePanel. This weight is designed for sample selection purposes.

**Study-Specific Post-Stratification Weights**

Once the sample has been selected and fielded, and all the study data are collected and made final, a post-stratification process is used to adjust for any survey non-response as well as any non-coverage or under- and over-sampling resulting from the study-specific sample design. Demographic and geographic distributions for the non-institutionalized, civilian population ages

18+ from the most recent CPS are used as benchmarks in this adjustment.

The following benchmark distributions are utilized for this post-stratification adjustment.

Gender (Male/Female)

Age (18–29, 30–44, 45–59, and 60+)

Race/Hispanic ethnicity (White/Non-Hispanic, Black/Non-Hispanic, Other/Non- Hispanic, 2+ Races/Non-Hispanic, Hispanic)

Education (Less than High School, High School, Some College, Bachelors and higher) Census Region (Northeast, Midwest, South, West)

Metropolitan Area (Yes, No) Internet Access (Yes, No)

Comparable distributions are calculated by using all completed cases from the field data (n = X). Since study sample sizes are typically too small to accommodate a complete cross-tabulation of all the survey variables with the benchmark variables, a raking procedure is used for the post- stratification weighting adjustment. Using the base weight as the starting weight, this procedure adjusts the sample data back to the selected benchmark proportions. Through an iterative convergence process, the weighted sample data are optimally fitted to the marginal distributions.

After this final post-stratification adjustment, the distribution of the calculated weights are examined to identify and, if necessary, trim outliers at the extreme upper and lower tails of the weight distribution. The post-stratified and trimmed weights are then scaled to the sum of the total sample size of all eligible respondents.

Attributions of Responsibility for Obesity

July 2011

- Questionnaire -

**[SP; PROMPT]**

**Thank you for agreeing to participate in the study. The purpose of this study is to gain a better understanding of how people think about health and community. If you decide to participate in this study, you may be asked to read a short message; you will also be asked to answer a series of questions. The study will take about 25 minutes to complete.**

**You may ask any questions about the research at any time. If you have questions about the research after you leave today, you should contact the Principal Investigator, Dr. Jeff Niederdeppe (607-255-9706;** [**jdn56@cornell.edu**](mailto:jdn56@cornell.edu)**). If you are not satisfied with response of the research team, have more questions, or want to talk with someone about your rights as a research participant, you should contact the Cornell University Institutional Review Board (IRB) at 607-255-5138 or access their website at** [**http://www.irb.cornell.edu**](http://www.irb.cornell.edu/)**. You may also report your concerns or complaints anonymously through Ethicspoint or by calling toll free at 1-866-293-3077. Ethicspoint is an independent organization that serves as a liaison between the University and the person bringing the complaint so that anonymity can be ensured.**

**I have read the above information, and have received answers to any questions I asked. I consent to take part in the study.**

**I AGREE TO PARTICIPATE I DECLINE TO PARTICIPATE**

(NOTE - only respondents who agree to participate will proceed to the study)

**TERMINATE IF DECLINE TO PARTICIPATE OR REFUSED AFTER PROMPT**

**RANDOMIZED CONDITION VARIABLE: DOV_MESSAGE**

**1=MESSAGE 1**

**2=MESSAGE 2**

**3=MESSAGE 3**

**4=MESSAGE 4**

**5=MESSAGE 5**

**6=MESSAGE 6**

**7=MESSAGE 7**

**8=MESSAGE 8**

**9=NO MESSAGE**

**USE ROTATION TO ASSIGN RESPONDENTS TO DOV_MESSAGE=1-9**

NOTE: Respondents will then be randomly assigned to one of eight conditions, or will be skipped through this section (the 9^th^ condition, a control group). Text that is listed in red **should not** be shown to respondents, including the headers at the top of every section.

**(MESSAGE 1 - EMPATHY, DEMOCRAT, LOW RESPONSIBILITY),** a story about Michele Wolfe who volunteers for the Democratic National Committee

**(MESSAGE 2 - RATIONAL, DEMOCRAT, LOW RESPONSIBILITY),** a story about Michele Wolfe who volunteers for the Democratic National Committee

**(MESSAGE 3 -** **EMPATHY, REPUBLICAN, LOW RESPONSIBILITY),** a story about Michele Wolfe who volunteers for the Republican National Committee

**(MESSAGE 4 -** **RATIONAL, REPUBLICAN, LOW RESPONSIBILITY),** a story about Michele Wolfe who volunteers for the Republican National Committee

**(MESSAGE 5 - EMPATHY, DEMOCRAT, HIGH RESPONSIBILITY),** a story about Michele Wolfe who volunteers for the Democratic National Committee

**(MESSAGE 6 - RATIONAL, DEMOCRAT, HIGH RESPONSIBILITY),** a story about Michele Wolfe who volunteers for the Democratic National Committee

**(MESSAGE 7 -** **EMPATHY, REPUBLICAN, HIGH RESPONSIBILITY),** a story about Michele Wolfe who volunteers for the Republican National Committee

**(MESSAGE 8 -** **RATIONAL, REPUBLICAN, HIGH RESPONSIBILITY),** a story about Michele Wolfe who volunteers for the Republican National Committee

**[IF DOV_MESSAGE=1-8]**

**We’d like to begin by asking you to view a short message. Once you click “Continue,” the text of the message will appear on your screen – please read along.**

The **(NO MESSAGE 9)** group will proceed directly to a subset of the questions starting on page 19, without the text listed above.

(NOTE – it would be optimal for message v1-v8 for the story to appear on the screen for some time before the “Continue” button appears, say 90 seconds or so)

**IF DOV_MESSAGE=1**

**[DISPLAY]**

**Message v1**

**While you are reading this message, try to imagine how Michele feels about what has happened and how it has affected her life. Try to feel the full impact of what Michelle has been through and how she feels as a result.**

**
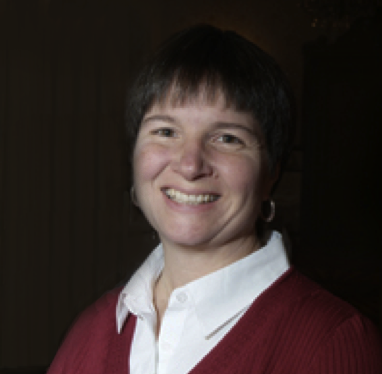
**

Michele, 41, was at high risk of developing diabetes and high blood pressure—probably due to a combination of genetics, her environment, and finances, she says. In her family, hearty, inexpensive foods are the suppertime staples. One night they have spaghetti and meatballs, the next night macaroni and cheese. Her grandmother cooks plenty of pork too. And not long ago, getting outdoors was also a risky proposition in her once crime-ridden, traffic-congested neighborhood with few safe parks and playgrounds.

Recently, however, Michele has dropped 11 pounds, and she didn’t even realize it.

“I haven’t been trying to lose weight, I guess it just happened. I haven’t changed my diet, gone to the gym, or tried to change my habits in any way. With two jobs and the time I spend volunteering for the local committee for the Democratic Party, there’s no time to count calories or prepare special ‘healthy’ meals. To tell you the truth, I’m really not concerned with my weight, but I guess I’m glad I lost it.”

Many people like Michele don’t have the time or energy to adopt major lifestyle changes. Instead, community organizations are doing what they can to help improve the health of people, who, like Michele, don’t place healthy diet and regular exercise at a very high priority.

One group, the Neighborhood Development Association (NDA), has helped to transform Michele’s neighborhood. She thinks these changes are responsible for her weight loss. NDA’s development of jogging-biking trails, public parks, and a new playground has increased residents’ opportunities for safe physical activity. They have also changed people’s daily routines.

“The neighborhood definitely looks nicer since NDA’s renovation. However, it has changed my commute. Because of all the green spaces there are less parking spots, I now walk about eight blocks on my way to work and on my way home.”

Thanks to NDA, Michele now lives in a true community. Here, she feels more comfortable getting out of the house, even if she’s not intending to exercise. This has helped Michele to improve her health, even though this is not one of her priorities.

“Juggling work, family, and finances, I don’t have the time or the energy to plan my meals ahead of time or squeeze in a workout. I’m worried about providing for my family, so I can’t be jogging around town.”

Despite Michele’s busy schedule, NDA has made some changes that have improved her diet, as well. NDA has brought a new supermarket and farmer’s market into the neighborhood. These resources improve the availability of fresh fruits and produce and make it easier for Michele and her neighbors to access cheap and healthy foods.

“I’m not about to spend more money for less food, even if it’s healthier, if we are not going to enjoy it. It just makes sense for us to eat things we like and save money at the same time. However, my family does like some types of vegetables, so, because they’re cheaper at the new supermarket and I pass it on my way home, I’m buying them a bit more often.”

“It’s the responsibility of the community to create a healthier neighborhood for the people living here,” Michele says. “I have other things to worry about – like paying the bills and feeding my family.”

Even though Michele hasn’t made her own health a priority, NDA is doing their part to positively influence her well-being.

**Message v2**

**IF DOV_MESSAGE=2**

**[DISPLAY]**

**While you are reading this message, try to take an objective perspective toward what is described. Try not to get caught up in how Michele feels; just remain objective and detached.**


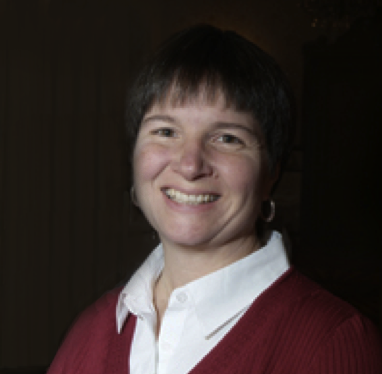
Michele, 41, was at high risk of developing diabetes and high blood pressure—probably due to a combination of genetics, her environment, and finances, she says. In her family, hearty, inexpensive foods are the suppertime staples. One night they have spaghetti and meatballs, the next night macaroni and cheese. Her grandmother cooks plenty of pork too. And not long ago, getting outdoors was also a risky proposition in her once crime-ridden, traffic-congested neighborhood with few safe parks and playgrounds.

Recently, however, Michele has dropped 11 pounds, and she didn’t even realize it.

“I haven’t been trying to lose weight, I guess it just happened. I haven’t changed my diet, gone to the gym, or tried to change my habits in any way. With two jobs and the time I spend volunteering for the local committee for the Democratic Party, there’s no time to count calories or prepare special ‘healthy’ meals. To tell you the truth, I’m really not concerned with my weight, but I guess I’m glad I lost it.”

Many people like Michele don’t have the time or energy to adopt major lifestyle changes. Instead, community organizations are doing what they can to help improve the health of people, who, like Michele, don’t place healthy diet and regular exercise at a very high priority.

One group, the Neighborhood Development Association (NDA), has helped to transform Michele’s neighborhood. She thinks these changes are responsible for her weight loss. NDA’s development of jogging-biking trails, public parks, and a new playground has increased residents’ opportunities for safe physical activity. They have also changed people’s daily routines.

“The neighborhood definitely looks nicer since NDA’s renovation. However, it has changed my commute. Because of all the green spaces there are less parking spots, I now walk about eight blocks on my way to work and on my way home.”

Thanks to NDA, Michele now lives in a true community. Here, she feels more comfortable getting out of the house, even if she’s not intending to exercise. This has helped Michele to improve her health, even though this is not one of her priorities.

“Juggling work, family, and finances, I don’t have the time or the energy to plan my meals ahead of time or squeeze in a workout. I’m worried about providing for my family, so I can’t be jogging around town.”

Despite Michele’s busy schedule, NDA has made some changes that have improved her diet, as well. NDA has brought a new supermarket and farmer’s market into the neighborhood. These resources improve the availability of fresh fruits and produce and make it easier for Michele and her neighbors to access cheap and healthy foods.

“I’m not about to spend more money for less food, even if it’s healthier, if we are not going to enjoy it. It just makes sense for us to eat things we like and save money at the same time. However, my family does like some types of vegetables, so, because they’re cheaper at the new supermarket and I pass it on my way home, I’m buying them a bit more often.”

“It’s the responsibility of the community to create a healthier neighborhood for the people living here,” Michele says. “I have other things to worry about – like paying the bills and feeding my family.”

Even though Michele hasn’t made her own health a priority, NDA is doing their part to positively influence her well-being.

**Message v3**

**IF DOV_MESSAGE=3**

**[DISPLAY]**

**While you are reading this message, try to imagine how Michele feels about what has happened and how it has affected her life. Try to feel the full impact of what Michelle has been through and how she feels as a result.**


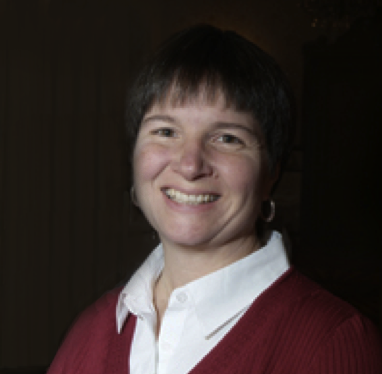
Michele, 41, was at high risk of developing diabetes and high blood pressure—probably due to a combination of genetics, her environment, and finances, she says. In her family, hearty, inexpensive foods are the suppertime staples. One night they have spaghetti and meatballs, the next night macaroni and cheese. Her grandmother cooks plenty of pork too. And not long ago, getting outdoors was also a risky proposition in her once crime-ridden, traffic-congested neighborhood with few safe parks and playgrounds.

Recently, however, Michele has dropped 11 pounds, and she didn’t even realize it.

“I haven’t been trying to lose weight, I guess it just happened. I haven’t changed my diet, gone to the gym, or tried to change my habits in any way. With two jobs and the time I spend volunteering for the local committee for the Republican Party, there’s no time to count calories or prepare special ‘healthy’ meals. To tell you the truth, I’m really not concerned with my weight, but I guess I’m glad I lost it.”

Many people like Michele don’t have the time or energy to adopt major lifestyle changes. Instead, community organizations are doing what they can to help improve the health of people, who, like Michele, don’t place healthy diet and regular exercise at a very high priority.

One group, the Neighborhood Development Association (NDA), has helped to transform Michele’s neighborhood. She thinks these changes are responsible for her weight loss. NDA’s development of jogging-biking trails, public parks, and a new playground has increased residents’ opportunities for safe physical activity. They have also changed people’s daily routines.

“The neighborhood definitely looks nicer since NDA’s renovation. However, it has changed my commute. Because of all the green spaces there are less parking spots, I now walk about eight blocks on my way to work and on my way home.”

Thanks to NDA, Michele now lives in a true community. Here, she feels more comfortable getting out of the house, even if she’s not intending to exercise. This has helped Michele to improve her health, even though this is not one of her priorities.

“Juggling work, family, and finances, I don’t have the time or the energy to plan my meals ahead of time or squeeze in a workout. I’m worried about providing for my family, so I can’t be jogging around town.”

Despite Michele’s busy schedule, NDA has made some changes that have improved her diet, as well. NDA has brought a new supermarket and farmer’s market into the neighborhood. These resources improve the availability of fresh fruits and produce and make it easier for Michele and her neighbors to access cheap and healthy foods.

“I’m not about to spend more money for less food, even if it’s healthier, if we are not going to enjoy it. It just makes sense for us to eat things we like and save money at the same time. However, my family does like some types of vegetables, so, because they’re cheaper at the new supermarket and I pass it on my way home, I’m buying them a bit more often.”

“It’s the responsibility of the community to create a healthier neighborhood for the people living here,” Michele says. “I have other things to worry about – like paying the bills and feeding my family.”

Even though Michele hasn’t made her own health a priority, NDA is doing their part to positively influence her well-being.

**Message v4**

**IF DOV_MESSAGE=4**

**[DISPLAY]**

**While you are reading this message, try to take an objective perspective toward what is described. Try not to get caught up in how Michele feels; just remain objective and detached.**


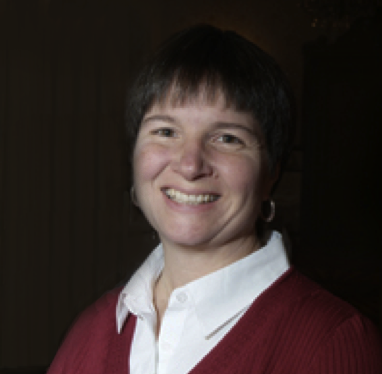
Michele, 41, was at high risk of developing diabetes and high blood pressure—probably due to a combination of genetics, her environment, and finances, she says. In her family, hearty, inexpensive foods are the suppertime staples. One night they have spaghetti and meatballs, the next night macaroni and cheese. Her grandmother cooks plenty of pork too. And not long ago, getting outdoors was also a risky proposition in her once crime-ridden, traffic-congested neighborhood with few safe parks and playgrounds.

Recently, however, Michele has dropped 11 pounds, and she didn’t even realize it.

“I haven’t been trying to lose weight, I guess it just happened. I haven’t changed my diet, gone to the gym, or tried to change my habits in any way. With two jobs and the time I spend volunteering for the local committee for the Republican Party, there’s no time to count calories or prepare special ‘healthy’ meals. To tell you the truth, I’m really not concerned with my weight, but I guess I’m glad I lost it.”

Many people like Michele don’t have the time or energy to adopt major lifestyle changes. Instead, community organizations are doing what they can to help improve the health of people, who, like Michele, don’t place healthy diet and regular exercise at a very high priority.

One group, the Neighborhood Development Association (NDA), has helped to transform Michele’s neighborhood. She thinks these changes are responsible for her weight loss. NDA’s development of jogging-biking trails, public parks, and a new playground has increased residents’ opportunities for safe physical activity. They have also changed people’s daily routines.

“The neighborhood definitely looks nicer since NDA’s renovation. However, it has changed my commute. Because of all the green spaces there are less parking spots, I now walk about eight blocks on my way to work and on my way home.”

Thanks to NDA, Michele now lives in a true community. Here, she feels more comfortable getting out of the house, even if she’s not intending to exercise. This has helped Michele to improve her health, even though this is not one of her priorities.

“Juggling work, family, and finances, I don’t have the time or the energy to plan my meals ahead of time or squeeze in a workout. I’m worried about providing for my family, so I can’t be jogging around town.”

Despite Michele’s busy schedule, NDA has made some changes that have improved her diet, as well. NDA has brought a new supermarket and farmer’s market into the neighborhood. These resources improve the availability of fresh fruits and produce and make it easier for Michele and her neighbors to access cheap and healthy foods.

“I’m not about to spend more money for less food, even if it’s healthier, if we are not going to enjoy it. It just makes sense for us to eat things we like and save money at the same time. However, my family does like some types of vegetables, so, because they’re cheaper at the new supermarket and I pass it on my way home, I’m buying them a bit more often.”

“It’s the responsibility of the community to create a healthier neighborhood for the people living here,” Michele says. “I have other things to worry about – like paying the bills and feeding my family.”

Even though Michele hasn’t made her own health a priority, NDA is doing their part to positively influence her well-being.

**Message v5**

**IF DOV_MESSAGE=5**

**[DISPLAY]**

**While you are reading this message, try to imagine how Michele feels about what has happened and how it has affected her life. Try to feel the full impact of what Michelle has been through and how she feels as a result.**


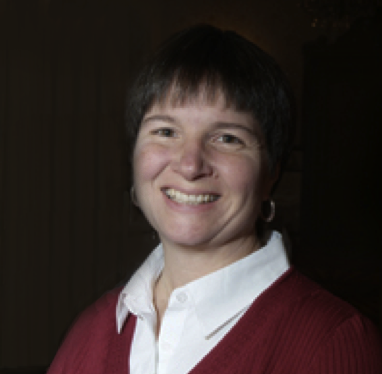
Michele, 41, was at high risk of developing diabetes and high blood pressure - probably due to a combination of genetics, personal choices, her environment, and finances, she says. In her family, hearty, inexpensive foods were the suppertime staples. One night they would have spaghetti and meatballs, the next night macaroni and cheese. Her grandmother cooked plenty of pork too. And when it came to exercise, getting outdoors was a risky proposition in crime-ridden, traffic-congested neighborhoods with few safe parks and playgrounds.

After struggling with her weight, she has dropped 11 pounds by counting calories, controlling portions, and adopting a diet that moves away from carbohydrates and toward fruits and vegetables.

Michele has always believed that it is her own personal responsibility to be healthy, but it hasn’t been easy.

“At first, I didn’t know how to cook a lot of healthy things,” she says. “The healthier stuff was always more expensive and less likely to fill me up,” says Michele. “With two jobs and the time I spend volunteering for the local committee for the Democratic Party, I don’t have a lot of time. There are so many cheap and delicious food options in my neighborhood that require little to no preparation at home, they were just easier.”

Fortunately, she’s gotten help. The Neighborhood Development Association (NDA) has helped transform Michele’s neighborhood, making her a big believer that one’s environment influences physical and emotional well-being. NDA has brought a new supermarket and farmer’s market into the neighborhood. These neighborhood resources improve the availability of fresh fruits and produce and make it easier for people like Michele to shop for healthy foods. In addition, NDA’s development of jogging-biking trails, public parks, and a new playground has increased residents’ opportunities for safe physical activity.

“I’m getting much more regular exercise than I used to,” she says. “I can push myself to get the quality of exercise I need. Physical activity is also built into my routine.”

Thanks to NDA, Michele now lives in a true community – where neighbors look out for each other and residents place importance on projects such as landscaping and recycling. Here, she feels comfortable getting out of the house and exercising outside – activities Michele sees as tremendously important for improving her health. This has helped Michele to develop healthier lifestyle habits.

“I look for the specials,” she says, “say eight peppers to a bag or the $1-a-bag special that week.”

Along the way, Michele has gotten her friends and family involved too. She and her co-workers now share recipes for all sorts of healthy foods, like spinach, squash, cabbage, and collard greens. Her children participate in the shopping and cooking too, Michele says, expanding their food repertoire while also keeping mom healthy.

“I won’t say it’s been easy – there have been many challenges along the way. Still, it’s my responsibility to keep myself and my family healthy and to not make excuses,” Michele says. “What NDA has done for the neighborhood has been a huge boost.”

With help from NDA, Michele is now on the path to better health.

**Message v6**

**IF DOV_MESSAGE=6**

**[DISPLAY]**

**While you are reading this message, try to take an objective perspective toward what is described. Try not to get caught up in how Michele feels; just remain objective and detached.**


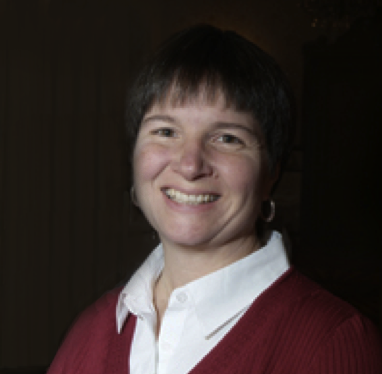
Michele, 41, was at high risk of developing diabetes and high blood pressure - probably due to a combination of genetics, personal choices, her environment, and finances, she says. In her family, hearty, inexpensive foods were the suppertime staples. One night they would have spaghetti and meatballs, the next night macaroni and cheese. Her grandmother cooked plenty of pork too. And when it came to exercise, getting outdoors was a risky proposition in crime-ridden, traffic-congested neighborhoods with few safe parks and playgrounds.

After struggling with her weight, she has dropped 11 pounds by counting calories, controlling portions, and adopting a diet that moves away from carbohydrates and toward fruits and vegetables.

Michele has always believed that it is her own personal responsibility to be healthy, but it hasn’t been easy.

“At first, I didn’t know how to cook a lot of healthy things,” she says. “The healthier stuff was always more expensive and less likely to fill me up,” says Michele. “With two jobs and the time I spend volunteering for the local committee for the Democratic Party, I don’t have a lot of time. There are so many cheap and delicious food options in my neighborhood that require little to no preparation at home, they were just easier.”

Fortunately, she’s gotten help. The Neighborhood Development Association (NDA) has helped transform Michele’s neighborhood, making her a big believer that one’s environment influences physical and emotional well-being. NDA has brought a new supermarket and farmer’s market into the neighborhood. These neighborhood resources improve the availability of fresh fruits and produce and make it easier for people like Michele to shop for healthy foods. In addition, NDA’s development of jogging-biking trails, public parks, and a new playground has increased residents’ opportunities for safe physical activity.

“I’m getting much more regular exercise than I used to,” she says. “I can push myself to get the quality of exercise I need. Physical activity is also built into my routine.”

Thanks to NDA, Michele now lives in a true community – where neighbors look out for each other and residents place importance on projects such as landscaping and recycling. Here, she feels comfortable getting out of the house and exercising outside – activities Michele sees as tremendously important for improving her health. This has helped Michele to develop healthier lifestyle habits.

“I look for the specials,” she says, “say eight peppers to a bag or the $1-a-bag special that week.”

Along the way, Michele has gotten her friends and family involved too. She and her co-workers now share recipes for all sorts of healthy foods, like spinach, squash, cabbage, and collard greens. Her children participate in the shopping and cooking too, Michele says, expanding their food repertoire while also keeping mom healthy.

“I won’t say it’s been easy – there have been many challenges along the way. Still, it’s my responsibility to keep myself and my family healthy and to not make excuses,” Michele says. “What NDA has done for the neighborhood has been a huge boost.”

With help from NDA, Michele is now on the path to better health.

**Message v7**

**IF DOV_MESSAGE=7**

**[DISPLAY]**

**While you are reading this message, try to imagine how Michelle feels about what has happened and how it has affected her life. Try to feel the full impact of what Michele has been through and how she feels as a result.**


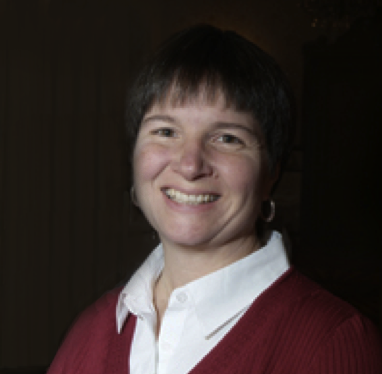
Michele, 41, was at high risk of developing diabetes and high blood pressure - probably due to a combination of genetics, personal choices, her environment, and finances, she says. In her family, hearty, inexpensive foods were the suppertime staples. One night they would have spaghetti and meatballs, the next night macaroni and cheese. Her grandmother cooked plenty of pork too. And when it came to exercise, getting outdoors was a risky proposition in crime-ridden, traffic-congested neighborhoods with few safe parks and playgrounds.

After struggling with her weight, she has dropped 11 pounds by counting calories, controlling portions, and adopting a diet that moves away from carbohydrates and toward fruits and vegetables.

Michele has always believed that it is her own personal responsibility to be healthy, but it hasn’t been easy.

“At first, I didn’t know how to cook a lot of healthy things,” she says. “The healthier stuff was always more expensive and less likely to fill me up,” says Michele. “With two jobs and the time I spend volunteering for the local committee for the Republican Party, I don’t have a lot of time. There are so many cheap and delicious food options in my neighborhood that require little to no preparation at home, they were just easier.”

Fortunately, she’s gotten help. The Neighborhood Development Association (NDA) has helped transform Michele’s neighborhood, making her a big believer that one’s environment influences physical and emotional well-being. NDA has brought a new supermarket and farmer’s market into the neighborhood. These neighborhood resources improve the availability of fresh fruits and produce and make it easier for people like Michele to shop for healthy foods. In addition, NDA’s development of jogging-biking trails, public parks, and a new playground has increased residents’ opportunities for safe physical activity.

“I’m getting much more regular exercise than I used to,” she says. “I can push myself to get the quality of exercise I need. Physical activity is also built into my routine.”

Thanks to NDA, Michele now lives in a true community – where neighbors look out for each other and residents place importance on projects such as landscaping and recycling. Here, she feels comfortable getting out of the house and exercising outside – activities Michele sees as tremendously important for improving her health. This has helped Michele to develop healthier lifestyle habits.

“I look for the specials,” she says, “say eight peppers to a bag or the $1-a-bag special that week.”

Along the way, Michele has gotten her friends and family involved too. She and her co-workers now share recipes for all sorts of healthy foods, like spinach, squash, cabbage, and collard greens. Her children participate in the shopping and cooking too, Michele says, expanding their food repertoire while also keeping mom healthy.

“I won’t say it’s been easy – there have been many challenges along the way. Still, it’s my responsibility to keep myself and my family healthy and to not make excuses,” Michele says. “What NDA has done for the neighborhood has been a huge boost.”

With help from NDA, Michele is now on the path to better health.

**Message v8**

**IF DOV_MESSAGE=8**

**[DISPLAY]**

**While you are reading this message, try to take an objective perspective toward what is described. Try not to get caught up in how Michele feels; just remain objective and detached.**


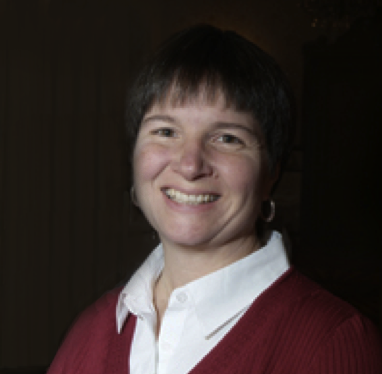
Michele, 41, was at high risk of developing diabetes and high blood pressure - probably due to a combination of genetics, personal choices, her environment, and finances, she says. In her family, hearty, inexpensive foods were the suppertime staples. One night they would have spaghetti and meatballs, the next night macaroni and cheese. Her grandmother cooked plenty of pork too. And when it came to exercise, getting outdoors was a risky proposition in crime-ridden, traffic-congested neighborhoods with few safe parks and playgrounds.

After struggling with her weight, she has dropped 11 pounds by counting calories, controlling portions, and adopting a diet that moves away from carbohydrates and toward fruits and vegetables.

Michele has always believed that it is her own personal responsibility to be healthy, but it hasn’t been easy.

“At first, I didn’t know how to cook a lot of healthy things,” she says. “The healthier stuff was always more expensive and less likely to fill me up,” says Michele. “With two jobs and the time I spend volunteering for the local committee for the Republican Party, I don’t have a lot of time. There are so many cheap and delicious food options in my neighborhood that require little to no preparation at home, they were just easier.”

Fortunately, she’s gotten help. The Neighborhood Development Association (NDA) has helped transform Michele’s neighborhood, making her a big believer that one’s environment influences physical and emotional well-being. NDA has brought a new supermarket and farmer’s market into the neighborhood. These neighborhood resources improve the availability of fresh fruits and produce and make it easier for people like Michele to shop for healthy foods. In addition, NDA’s development of jogging-biking trails, public parks, and a new playground has increased residents’ opportunities for safe physical activity.

“I’m getting much more regular exercise than I used to,” she says. “I can push myself to get the quality of exercise I need. Physical activity is also built into my routine.”

Thanks to NDA, Michele now lives in a true community – where neighbors look out for each other and residents place importance on projects such as landscaping and recycling. Here, she feels comfortable getting out of the house and exercising outside – activities Michele sees as tremendously important for improving her health. This has helped Michele to develop healthier lifestyle habits.

“I look for the specials,” she says, “say eight peppers to a bag or the $1-a-bag special that week.”

Along the way, Michele has gotten her friends and family involved too. She and her co-workers now share recipes for all sorts of healthy foods, like spinach, squash, cabbage, and collard greens. Her children participate in the shopping and cooking too, Michele says, expanding their food repertoire while also keeping mom healthy.

“I won’t say it’s been easy – there have been many challenges along the way. Still, it’s my responsibility to keep myself and my family healthy and to not make excuses,” Michele says. “What NDA has done for the neighborhood has been a huge boost.”

With help from NDA, Michele is now on the path to better health.

**Thought Listing**

**[TEXT BOXES]**

Q1.

**MESSAGES v1-v8:**

**[IF DOV_MESSAGE=1-8]**

**I’d like for you to list five thoughts that came into your mind as you were reading the story. Just try to remember the thoughts that crossed your mind while you were reading the story. Please try to write out sentence-length descriptions of each thought.**

**NO MESSAGE 9:**

**[IF DOV_MESSAGE=9]**

**First, I’d like for you to list the first five thoughts that come to your mind when you think of the words “overweight” and “obesity.” Just write down the thoughts that cross your mind while you think about the words. Please try to write out sentence-length descriptions of each thought.**

(Respondents are given 1 minute to list up to five thoughts by typing them into text boxes. After 1 minute, a box should appear that will allow them to move to the next part of the survey).

**Q1_01** = verbatim text from first thought

**Q2_02** = verbatim text from second thought

**Q3_03** = verbatim text from third thought

**(see ThoughtsCodebook for details on how this information was quantified for the analysis)**

**Manipulation Check** (order to be randomized) **(MESSAGES 1-8 ONLY)**

**IF DOV_MESSAGE=1-8**

**[GRID; SP; RANDOMIZE]**

Q2. **How much do you agree or disagree with the following statements?**

1 2 3 4 5

Strongly disagree Disagree Neither Disagree/Agree Agree Strongly Agree

1. The story emphasized the role of Michele’s personal decisions in her weight loss. **Q2_A**
2. The story emphasized the role of Michele’s neighborhood in her weight loss. **Q2_B**
3. The story suggested that Michele is personally responsible for losing weight. **Q2_C**
4. The story suggested that her society is responsible for helping Michele to lose weight. **Q2_D**
5. The story suggested that losing weight is under Michele’s control. **Q2_E**
6. The story suggested that weight loss is outside of Michele’s control. **Q2_F**

**IF DOV_MESSAGE=1-8**

**[GRID; SP]**

Q3. How controllable was the reason for Michele’s struggle with her weight? **Q3_1**

1 2 3 4 5

Very Somewhat Neither Somewhat Very Uncontrollable Uncontrollable Uncontrollable or Controllable Controllable

Controllable

**Emotional Responses to Character (MESSAGES 1-8 ONLY; order to be randomized)**

**IF DOV_MESSAGE=1-8**

**[GRID; SP; RANDOMIZE]**

Q4. **Thinking about the message you read…**

1 2 3 4

hardly any just some a good amount a great deal

1. How much do you blame Michele for her circumstances? **Q4_A**
2. How much anger do you feel toward Michele? **Q4_B**
3. How much pity do you feel toward Michele? **Q4_C**
4. How much sympathy do you have for Michele? **Q4_D**

**Policy Beliefs (ALL; order to be randomized)**

**[GRID; SP; RANDOMIZE]**

Q5. **Many different ideas have been proposed to address the number of people who are overweight in the US. Please indicate whether you strongly oppose, oppose, neither support nor oppose, support, or strongly support each of the following policies.**

1 2 3 4 5

strongly oppose oppose neither support strongly support

1. Have zoning laws requiring that all new residential and commercial developments include sidewalks and other safe paths to encourage physical activity **Q5_A**
2. Require restaurants to list the calorie count and fat content of all items on their menus **Q5_B**
3. Require grocers to add a surcharge to high-sugar, high-fat foods and use the revenues to reduce their prices for fresh fruits and vegetables **Q5_C**
4. Create a government-funded public education campaign on healthy eating and exercise **Q5_D**
5. Provide funding to public schools to make fresh fruit, vegetables, and low-fat milk available for free at school lunches. **Q5_E**
6. Increase options for affordable transportation to parks and open spaces by discounting public transit, altering or expanding school bus routes, and providing incentives for ride sharing. **Q5_F**
7. Break down barriers to physical activity through joint-use agreements, where spaces for physical activity are required to be made available to the entire community. **Q5_G**
8. Support the sale of local foods across the community by offering incentives and encouraging the establishment of farmers’ markets. **Q5_H**

**Attributions of Responsibility – Causes (ALL; order to be randomized)**

**[GRID; SP; RANDOMIZE]**

Q6**. I am now going to list a series of explanations some people give about why many Americans are overweight. In response to each statement, please tell us how much you agree with it as an explanation for why many Americans are overweight.**

1 2 3 4 5

Strongly disagree Disagree Neither Disagree/Agree Agree Strongly Agree

1. Healthy food is too expensive for many people **Q6_A**
2. There are not enough healthy food options in restaurants and supermarkets **Q6_B**
3. There are too many unhealthy food options in restaurants and supermarkets **Q6_C**
4. There are not enough safe and affordable places for people to exercise **Q6_D**
5. Most people lack the willpower to diet regularly **Q6_E**
6. Most people lack the willpower to exercise regularly **Q6_F**
7. Most overweight people lack self control **Q6_G**
8. Most overweight people don’t view their weight as a problem **Q6_H**

**Attributions of Responsibility – Solutions (ALL; order to be randomized)**

**[GRID; SP; RANDOMIZE]**

Q7. **Whatever the causes of obesity, please tell me whether you think each of the following groups bears hardly any, just some, a good amount, or a great deal of responsibility for addressing the number of people who are overweight in the US.**

1 2 3 4

hardly any just some a good amount a great deal

1. Individuals in their choice of diet and lack of exercise **Q7_A**
2. Local government **Q7_B**
3. Federal government **Q7_C**
4. Community organizations **Q7_D**
5. Employers **Q7_E**
6. Manufacturers of unhealthy foods **Q7_F**
7. Restaurants that serve unhealthy foods **Q7_G**
8. Supermarkets that sell unhealthy foods **Q7_H**

**Response to the Message (order to be randomized)**

**IF DOV_MESSAGE=1-8**

**[GRID; SP; RANDOMIZE]**

Q8. **Now we’d like to ask you a few more questions about the message you read. How much do you agree or disagree with the following statements? (MESSAGES 1-8 ONLY)**

1 2 3 4 5

Strongly Disagree Disagree Neither Disagree/Agree Agree Strongly Agree

1. This message was very entertaining **Q8_A**
2. I enjoyed reading this message **Q8_B**
3. The message was personally relevant to me **Q8_C**
4. The message said something important to me **Q8_D**
5. The message was very interesting to me **Q8_E**

**IF DOV_MESSAGE=9**

**[GRID; SP; RANDOMIZE]**

Q9. **How much do you agree or disagree with the following statements about the topic of obesity?** **(NO MESSAGE 9 ONLY)**

1 2 3 4 5

Strongly Disagree Disagree Neither Disagree/Agree Agree Strongly Agree

1. This topic is very entertaining **Q9_A**
2. I enjoy thinking about this topic **Q9_B**
3. This topic is personally relevant to me **Q9_C**
4. This topic is something important to me **Q9_D**
5. This topic is very interesting to me **Q9_E**

**Empathy (MESSAGES 1-8 ONLY; order to be randomized)**

**IF DOV_MESSAGE=1-8**

**[GRID; SP; RANDOMIZE]**

Q10. **How much do you agree or disagree with the following statements about the message?**

1 2 3 4 5

Strongly Disagree Disagree Neither Disagree/Agree Agree Strongly Agree

1. I was touched by Michele’s situation. **Q10_A**
2. I felt upset for those who suffer from the problem described in the message. **Q10_B**
3. When I was reading the message, I felt sad for Michele. **Q10_C**
4. I do not understand how people could get themselves into a difficult situation like the one described. **Q10_D**
5. The message just seemed illogical to me. **Q10_E**
6. I am baffled by people who get into situations like the one described. **Q10_F**

**Realism with Self and Typical Person (MESSAGES 1-8 ONLY; order to be randomized)**

**IF DOV_MESSAGE=1-8**

**[GRID; SP; RANDOMIZE]**

Q11. **How much do you agree or disagree with the following statements about the message?**

1 2 3 4 5

Strongly Disagree Disagree Neither Disagree/Agree Agree Strongly Agree

1. If this were to happen to me it would happen this way. **Q11_A**
2. This story describes a situation that could really happen to me. **Q11_B**
3. If this were to happen to me it would make sense. **Q11_C**
4. If this were to happen to the typical person it would happen this way. **Q11_D**
5. This story describes a situation that could really happen to a typical person. **Q11_E**
6. If this were to happen to the typical person it would make sense. **Q11_F**

**Similarity with Real and Ideal Self (MESSAGES 1-8 ONLY; order to be randomized)**

**IF DOV_MESSAGE=1-8**

**[GRID; SP; RANDOMIZE]**

Q12. **How much do you agree or disagree with the following statements about the message?**

1 2 3 4 5

Strongly Disagree Disagree Neither Disagree/Agree Agree Strongly Agree

1. Michele has values that are similar to the values I actually practice. **Q12_A**
2. What the story says about Michele shows that she seems a lot like the person I actually am. **Q12_B**
3. My real self is similar to Michele. **Q12_C**
4. Michele has values that are like the values I would ideally wish to practice. **Q12_D**
5. What the story says about Michele shows that she seems a lot like the person I ideally would like to be. **Q12_E**
6. My ideal self is similar to Michele. **Q12_F**

**Inclusion of Other in the Self Scale (IOS)**

**[SP] Q13**

Q13. **Below are seven pictures that depict possible ways of viewing the relationship between two people. Please click on the letter corresponding to the picture that best indicates the extent to which you feel a connection with the person in the story you read.**

**[SHOW IMAGES VERTICALLY]**

1. a

2. b

3. c

4. d

5. e

6. f

7. g

**
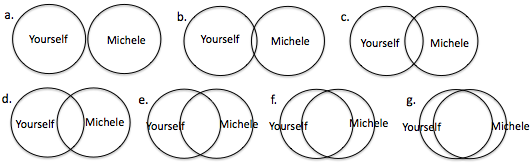
**

**[SP] Q14**

Q14**. “Your Real Self” is yourself as actually you are. Below are seven pictures that depict possible ways of viewing the relationship between two people. Please click on the letter corresponding to the picture that best indicates the extent to which the person in the story connects to you as you ACTUALLY are.**

**[SHOW IMAGES VERTICALLY]**

1. a

2. b

3. c

4. d

5. e

6. f

7. g

**
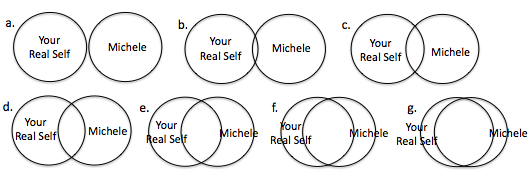
**

**[SP] Q15**

Q15. **“Your Ideal Self” is yourself as you would like to be. Below are seven pictures that depict possible ways of viewing the relationship between two people. Please click on the letter corresponding to the picture that best indicates the extent to which the person in the story connects to you as you are IDEALLY likely to be.**

**[SHOW IMAGES VERTICALLY]**

1. a

2. b

3. c

4. d

5. e

6. f

7. g

**
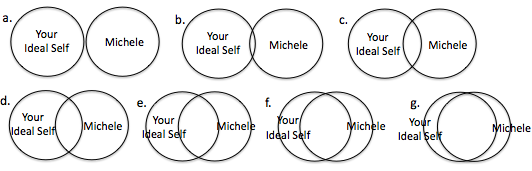
**

**Beliefs about Disparities by Income, Education and Race (ALL RESPONDENTS; randomize order)**

**[GRID; SP; RANDOMIZE]**

Q16. **Now I’d like to ask you some more general questions about the health of different groups of people.**

1 2 3

Better health overall Same health overall Worse health overall

1. Do you think **African Americans** tend to have better health overall, the same health overall, or worse health overall than whites? **Q16_A**
2. Do you think **those with a college degree** tend to have better health overall, the same health overall, or worse health overall than those without a college degree? **Q16_B**
3. Do you think **the poor** tend to have better health overall, the same health overall, or worse health overall than the middle class? **Q16_C**

**Intentions to Engage in Diet and Exercise Behaviors (ALL)**

**[GRID; SP]**

Q17. **Next, I’d like to ask you some questions about your thoughts about diet and exercise.**

1 2 3 4 5

Very unlikely unlikely neither likely nor unlikely likely very likely

1. How likely is it that you will have five or more servings of fruits and vegetables most days in the next year? **Q17_A**
2. How likely is it that you will exercise at least three times in most weeks over the next year? **Q17_B**
3. How likely is it that you will control your diet to lose weight in the next year? **Q17_C**

**Opposition to Inequality (ALL; order to be randomized)**

**[GRID; SP; RANDOMIZE]**

Q22. **How much do you agree or disagree with the following statements?**

1 2 3 4 5

Strongly Disagree Disagree Neither Disagree/Agree Agree Strongly Agree

1. Group equality should be our ideal. **Q22_A**
2. We should do what we can to equalize conditions for different groups. **Q22_B**
3. Increased social equality would be a good thing. **Q22_C**
4. It would be good if all groups could be equal. **Q22_D**
5. We would have fewer problems if we treated different groups more equally. **Q22_E**
6. All groups should be given an equal chance in life. **Q22_F**
7. No one group should dominate in society. **Q22_G**
8. We should strive to make incomes more equal. **Q22_H**

**Demographics (ALL)**

**[DISPLAY]**

**We’re almost finished – I have just a few more questions.**

**[SP] Q23_1**

Q23. Generally speaking, do you consider yourself to be a Democrat, a Republican, an Independent, or something else?

Republican Democrat Independent Something else

**[GRID; SP] Q24_1**

Q24. We hear a lot of talk these days about liberals and conservatives. On a scale from 1 to 7, where 1 means very liberal, 4 means moderate or middle of the road, and 7 means very conservative, which of the following do you usually think of yourself as?

1 2 3 4 5 6 7

Very liberal Moderate Very conservative

**[NUMBER BOXES; FEET RANGE 2-7; INCHES RANGE 0-11]**

Q25. How tall are you without shoes? Please type in the number of feet and inches separately. For example, if you are 6'0" tall, type 6 in the feet box and 0 in the inches box.

Feet (Number box with range 2 to 7) **Q25_Feet**

Inches (Number box with range 0 to 11) **Q25_Inches**

**[NUMBER BOX; RANGE 50-500]**

Q26. How much do you weigh without shoes? **Q26**

Pounds (Number box with range 50 to 500)

**[DISPLAY]**

**Thank you very much for your participation!**

**Supplemental Variables**

The table below shows the name and description of each of the supplemental, demographic, and other profile variables delivered to the client.

Variable Name Variable Description

PPAGE Age (continuous; range 18 to 99)

PPAGECAT Age - 7 Categories

PPAGECT4 Age - 4 Categories

PPEDUC Education (Highest Degree Received) PPEDUCAT Education (Categorical)

PPETHM Race / Ethnicity PPGENDER Gender PPHHHEAD Household Head PPHHSIZE Household Size PPHOUSE Housing Type PPINCIMP Household Income PPMARIT Marital Status PPMSACAT MSA Status

PPREG4 Region 4 - Based on State of Residence PPREG9 Region 9 - Based on State of Residence PPRENT Ownership Status of Living Quarters

PPT01 Presence of Household Members - Children 0-2

PPT25 Presence of Household Members - Children 2-5

PPT612 Presence of Household Members - Children 6-12

PPT1317 Presence of Household Members - Children 13-17

PPT18OV Presence of Household Members - Adults 18+ PPWORK Current Employment Status

PPNET HH Internet Access

**ppagecat Age - 7 Categories**

|  | | Frequency | Percent | Valid  Percent | Cumulative  Percent |
| --- | --- | --- | --- | --- | --- |
| Valid | 1 18-24 | 78 | 10.9 | 10.9 | 10.9 |
|  | 2 25-34 | 135 | 18.8 | 18.8 | 29.7 |
|  | 3 35-44 | 130 | 18.0 | 18.0 | 47.7 |
|  | 4 45-54 | 132 | 18.4 | 18.4 | 66.1 |
|  | 5 55-64 | 125 | 17.4 | 17.4 | 83.5 |
|  | 6 65-74 | 82 | 11.4 | 11.4 | 94.9 |
|  | 7 75+ | 36 | 5.1 | 5.1 | 100.0 |
|  | Total | 718 | 100.0 | 100.0 |  |

**ppagect4 Age - 4 Categories**

|  | | Frequency | Percent | Valid  Percent | Cumulative  Percent |
| --- | --- | --- | --- | --- | --- |
| Valid | 1 18-29 | 159 | 22.1 | 22.1 | 22.1 |
|  | 2 30-44 | 184 | 25.6 | 25.6 | 47.7 |
|  | 3 45-59 | 198 | 27.6 | 27.6 | 75.3 |
|  | 4 60+ | 177 | 24.7 | 24.7 | 100.0 |
|  | Total | 718 | 100.0 | 100.0 |  |

**PPEDUC Education (Highest Degree Received)**

|  | | Frequency | Percent | Valid  Percent | Cumulative  Percent |
| --- | --- | --- | --- | --- | --- |
| Valid | 1 No formal education | 5 | .7 | .7 | .7 |
|  | 2 1st, 2nd, 3rd, or 4th grade | 3 | .3 | .3 | 1.1 |
|  | 3 5th or 6th grade | 8 | 1.1 | 1.1 | 2.2 |
|  | 4 7th or 8th grade | 11 | 1.5 | 1.5 | 3.7 |
|  | 5 9th grade | 13 | 1.8 | 1.8 | 5.5 |
|  | 6 10th grade | 18 | 2.5 | 2.5 | 8.0 |
|  | 7 11th grade | 6 | .8 | .8 | 8.8 |
|  | 8 12th grade NO DIPLOMA | 32 | 4.4 | 4.4 | 13.2 |
|  | 9 HIGH SCHOOL GRADUATE - high school | 220 | 30.6 | 30.6 | 43.8 |
|  | DIPLOMA or the equivalent |  |  |  |  |
|  | 10 Some college, no degree | 152 | 21.2 | 21.2 | 65.0 |
|  | 11 Associate degree | 51 | 7.1 | 7.1 | 72.1 |
|  | 12 Bachelors degree | 116 | 16.2 | 16.2 | 88.3 |
|  | 13 Masters degree | 61 | 8.5 | 8.5 | 96.8 |
|  | 14 Professional or Doctorate  degree | 23 | 3.2 | 3.2 | 100.0 |

Total

718 100.0 100.0

**PPEDUCAT Education (Categorical)**

|  | | Frequency | Percent | Valid  Percent | Cumulative  Percent |
| --- | --- | --- | --- | --- | --- |
| Valid | 1 Less than high school | 95 | 13.2 | 13.2 | 13.2 |
|  | 2 High school | 220 | 30.6 | 30.6 | 43.8 |
|  | 3 Some college | 203 | 28.3 | 28.3 | 72.1 |
|  | 4 Bachelor's degree or higher | 200 | 27.9 | 27.9 | 100.0 |
|  | Total | 718 | 100.0 | 100.0 |  |

**PPETHM Race / Ethnicity**

|  | | Frequency | Percent | Valid  Percent | Cumulative  Percent |
| --- | --- | --- | --- | --- | --- |
| Valid | 1 White, Non-Hispanic | 488 | 67.9 | 67.9 | 67.9 |
|  | 2 Black, Non-Hispanic | 83 | 11.6 | 11.6 | 79.5 |
|  | 3 Other, Non-Hispanic | 40 | 5.6 | 5.6 | 85.1 |
|  | 4 Hispanic | 98 | 13.7 | 13.7 | 98.8 |
|  | 5 2+ Races, Non-Hispanic | 8 | 1.2 | 1.2 | 100.0 |
|  | Total | 718 | 100.0 | 100.0 |  |

**PPGENDER Gender**

|  | |  |  | Valid | Cumulative |
| --- | --- | --- | --- | --- | --- |
|  |  | Frequency | Percent | Percent | Percent |
| Valid | 1 Male | 347 | 48.3 | 48.3 | 48.3 |
|  | 2 Female | 371 | 51.7 | 51.7 | 100.0 |
|  | Total | 718 | 100.0 | 100.0 |  |

**PPHHHEAD Household Head**

|  | |  |  | Valid | Cumulative |
| --- | --- | --- | --- | --- | --- |
|  |  | Frequency | Percent | Percent | Percent |
| Valid | 0 No | 160 | 22.3 | 22.3 | 22.3 |
|  | 1 Yes | 558 | 77.7 | 77.7 | 100.0 |
|  | Total | 718 | 100.0 | 100.0 |  |

**PPHHSIZE Household Size**

|  | | Frequency | Percent | Valid  Percent | Cumulative  Percent |
| --- | --- | --- | --- | --- | --- |
| Valid | 1 | 140 | 19.5 | 19.5 | 19.5 |
|  | 2 | 251 | 35.0 | 35.0 | 54.5 |
|  | 3 | 112 | 15.6 | 15.6 | 70.1 |
|  | 4 | 113 | 15.7 | 15.7 | 85.8 |
|  | 5 | 52 | 7.2 | 7.2 | 93.1 |
|  | 6 | 27 | 3.8 | 3.8 | 96.8 |
|  | 7 | 8 | 1.1 | 1.1 | 98.0 |
|  | 8 | 6 | .8 | .8 | 98.8 |
|  | 9 | 5 | .6 | .6 | 99.4 |
|  | 10 | 2 | .2 | .2 | 99.7 |
|  | 15 | 2 | .3 | .3 | 100.0 |
|  | Total | 718 | 100.0 | 100.0 |  |

**PPHOUSE Housing Type**

Valid

1 A one-family house detached

Frequency Percent

Valid

Percent

Cumulative

Percent

from any other house

2 A one-family house attached to one or more houses

3 A building with 2 or more apartments

4 A mobile home

5 Boat, RV, van, etc. Total

489 68.1 68.1 68.1

68 9.5 9.5 77.6

129 18.0 18.0 95.6

29 4.1 4.1 99.7

2 .3 .3 100.0

718 100.0 100.0

**PPINCIMP Household Income**

|  | | Frequency | Percent | Valid  Percent | Cumulative  Percent |
| --- | --- | --- | --- | --- | --- |
| Valid | 1 Less than $5,000 | 18 | 2.5 | 2.5 | 2.5 |
|  | 2 $5,000 to $7,499 | 11 | 1.6 | 1.6 | 4.1 |
|  | 3 $7,500 to $9,999 | 23 | 3.1 | 3.1 | 7.2 |
|  | 4 $10,000 to $12,499 | 27 | 3.8 | 3.8 | 11.0 |
|  | 5 $12,500 to $14,999 | 25 | 3.5 | 3.5 | 14.5 |
|  | 6 $15,000 to $19,999 | 31 | 4.3 | 4.3 | 18.8 |
|  | 7 $20,000 to $24,999 | 37 | 5.1 | 5.1 | 24.0 |
|  | 8 $25,000 to $29,999 | 43 | 6.0 | 6.0 | 30.0 |
|  | 9 $30,000 to $34,999 | 39 | 5.5 | 5.5 | 35.5 |
|  | 10 $35,000 to $39,999 | 40 | 5.6 | 5.6 | 41.0 |
|  | 11 $40,000 to $49,999 | 56 | 7.8 | 7.8 | 48.8 |
|  | 12 $50,000 to $59,999 | 63 | 8.7 | 8.7 | 57.5 |
|  | 13 $60,000 to $74,999 | 60 | 8.4 | 8.4 | 65.9 |
|  | 14 $75,000 to $84,999 | 47 | 6.5 | 6.5 | 72.4 |
|  | 15 $85,000 to $99,999 | 39 | 5.5 | 5.5 | 77.9 |
|  | 16 $100,000 to $124,999 | 78 | 10.9 | 10.9 | 88.8 |
|  | 17 $125,000 to $149,999 | 43 | 6.0 | 6.0 | 94.8 |
|  | 18 $150,000 to $174,999 | 19 | 2.7 | 2.7 | 97.5 |
|  | 19 $175,000 or more | 18 | 2.5 | 2.5 | 100.0 |
|  | Total | 718 | 100.0 | 100.0 |  |

**PPMARIT Marital Status**

|  | | Frequency | Percent | Valid  Percent | Cumulative  Percent |
| --- | --- | --- | --- | --- | --- |
| Valid | 1 Married | 373 | 52.0 | 52.0 | 52.0 |
|  | 2 Widowed | 35 | 4.9 | 4.9 | 56.8 |
|  | 3 Divorced | 78 | 10.8 | 10.8 | 67.7 |
|  | 4 Separated | 16 | 2.2 | 2.2 | 69.9 |
|  | 5 Never married | 153 | 21.3 | 21.3 | 91.2 |
|  | 6 Living with partner | 63 | 8.8 | 8.8 | 100.0 |
|  | Total | 718 | 100.0 | 100.0 |  |

**PPMSACAT MSA Status**

|  | |  |  | Valid | Cumulative |
| --- | --- | --- | --- | --- | --- |
|  |  | Frequency | Percent | Percent | Percent |
| Valid | 0 Non-Metro | 116 | 16.2 | 16.2 | 16.2 |
|  | 1 Metro | 602 | 83.8 | 83.8 | 100.0 |
|  | Total | 718 | 100.0 | 100.0 |  |

**PPREG4 Region 4 - Based on State of Residence**

|  | | Frequency | Percent | Valid  Percent | Cumulative  Percent |
| --- | --- | --- | --- | --- | --- |
| Valid | 1 Northeast | 132 | 18.4 | 18.4 | 18.4 |
|  | 2 Midwest | 156 | 21.7 | 21.7 | 40.2 |
|  | 3 South | 265 | 36.9 | 36.9 | 77.1 |
|  | 4 West | 165 | 22.9 | 22.9 | 100.0 |
|  | Total | 718 | 100.0 | 100.0 |  |

**ppreg9 Region 9 - Based on State of Residence**

|  | | Frequency | Percent | Valid  Percent | Cumulative  Percent |
| --- | --- | --- | --- | --- | --- |
| Valid | 1 New England | 32 | 4.5 | 4.5 | 4.5 |
|  | 2 Mid-Atlantic | 100 | 13.9 | 13.9 | 18.4 |
|  | 3 East-North Central | 94 | 13.1 | 13.1 | 31.6 |
|  | 4 West-North Central | 62 | 8.6 | 8.6 | 40.2 |
|  | 5 South Atlantic | 146 | 20.3 | 20.3 | 60.5 |
|  | 6 East-South Central | 42 | 5.9 | 5.9 | 66.4 |
|  | 7 West-South Central | 77 | 10.7 | 10.7 | 77.1 |
|  | 8 Mountain | 47 | 6.5 | 6.5 | 83.6 |
|  | 9 Pacific | 118 | 16.4 | 16.4 | 100.0 |
|  | Total | 718 | 100.0 | 100.0 |  |

**PPRENT Ownership Status of Living Quarters**

|  | | Frequency | Percent | Valid  Percent | Cumulative  Percent |
| --- | --- | --- | --- | --- | --- |
| Valid | 1 Owned or being bought by you or someone in your | 494 | 68.8 | 68.8 | 68.8 |
|  | household |  |  |  |  |
|  | 2 Rented for cash | 207 | 28.8 | 28.8 | 97.6 |
|  | 3 Occupied without  payment of cash rent | 17 | 2.4 | 2.4 | 100.0 |

Total

718 100.0 100.0

**PPT01 Presence of Household Members - Children 0-2**

|  | | Frequency | Percent | Valid  Percent | Cumulative  Percent |
| --- | --- | --- | --- | --- | --- |
| Valid | 0 | 677 | 94.3 | 94.3 | 94.3 |
|  | 1 | 35 | 4.9 | 4.9 | 99.2 |
|  | 2 | 6 | .8 | .8 | 100.0 |
|  | Total | 718 | 100.0 | 100.0 |  |

**PPT25 Presence of Household Members - Children 2-5**

|  | | Frequency | Percent | Valid  Percent | Cumulative  Percent |
| --- | --- | --- | --- | --- | --- |
| Valid | 0 | 625 | 87.1 | 87.1 | 87.1 |
|  | 1 | 65 | 9.0 | 9.0 | 96.1 |
|  | 2 | 28 | 3.9 | 3.9 | 100.0 |
|  | Total | 718 | 100.0 | 100.0 |  |

**PPT612 Presence of Household Members - Children 6-12**

|  | | Frequency | Percent | Valid  Percent | Cumulative  Percent |
| --- | --- | --- | --- | --- | --- |
| Valid | 0 | 606 | 84.3 | 84.3 | 84.3 |
|  | 1 | 64 | 9.0 | 9.0 | 93.3 |
|  | 2 | 43 | 6.0 | 6.0 | 99.3 |
|  | 3 | 5 | .7 | .7 | 100.0 |
|  | Total | 718 | 100.0 | 100.0 |  |

**PPT1317 Presence of Household Members - Children 13-17**

|  | | Frequency | Percent | Valid  Percent | Cumulative  Percent |
| --- | --- | --- | --- | --- | --- |
| Valid | 0 | 605 | 84.2 | 84.2 | 84.2 |
|  | 1 | 83 | 11.5 | 11.5 | 95.7 |
|  | 2 | 25 | 3.4 | 3.4 | 99.2 |
|  | 3 | 5 | .7 | .7 | 99.8 |
|  | 4 | 1 | .2 | .2 | 100.0 |
|  | Total | 718 | 100.0 | 100.0 |  |

**PPT18OV Presence of Household Members - Adults 18+**

|  | | Frequency | Percent | Valid  Percent | Cumulative  Percent |
| --- | --- | --- | --- | --- | --- |
| Valid | 1 | 151 | 21.0 | 21.0 | 21.0 |
|  | 2 | 380 | 52.9 | 52.9 | 73.9 |
|  | 3 | 118 | 16.5 | 16.5 | 90.4 |
|  | 4 | 45 | 6.2 | 6.2 | 96.6 |
|  | 5 | 19 | 2.7 | 2.7 | 99.2 |
|  | 7 | 3 | .4 | .4 | 99.6 |
|  | 9 | 2 | .3 | .3 | 99.9 |
|  | 11 | 0 | .1 | .1 | 100.0 |
|  | Total | 718 | 100.0 | 100.0 |  |

**PPWORK Current Employment Status**

Valid

1 Working - as a paid

Frequency Percent

Valid

Percent

Cumulative

Percent

employee

2 Working - self-employed

3 Not working - on temporary layoff from a job

4 Not working - looking for work

5 Not working - retired

6 Not working - disabled

7 Not working - other

Total

366 50.9 50.9 50.9

47 6.6 6.6 57.5

9 1.3 1.3 58.8

63 8.7 8.7 67.6

116 16.2 16.2 83.8

59 8.2 8.2 92.0

58 8.0 8.0 100.0

718 100.0 100.0

**PPNET HH Internet Access**

|  | |  |  | Valid | Cumulative |
| --- | --- | --- | --- | --- | --- |
|  |  | Frequency | Percent | Percent | Percent |
| Valid | 0 No | 180 | 25.0 | 25.0 | 25.0 |
|  | 1 Yes | 538 | 75.0 | 75.0 | 100.0 |
|  | Total | 718 | 100.0 | 100.0 |  |
