# Supplementary material for: Acknowledging Individual Responsibility while Emphasizing Social Determinants in Narratives to Promote Obesity-Reducing Public Policy: A Randomized Experiment
Source: PLoS One. 2015 Feb 23;10(2):e0117565. doi: 10.1371/journal.pone.0117565 (PMC4338108; doi:10.1371/journal.pone.0117565)
Supplement: S1 Thought Codebook — (DOC) [file pone.0117565.s008.doc]

**Obesity Responsibility Study Thought Listing Codes (REVISED JUNE 2011)**

**INSTRUCTIONS:**

1.) Read through this codebook carefully

2.) Read each thought and take particular note of the type of language used by the respondent and types of attributions that people make for the causes or solutions for obesity.

3.) Provide THREE codes for each thought. Each of these is a YES (1) or NO (0) question.

**GENERAL NOTES:**

On occasion, respondents will make statements that are not causes or solutions for obesity. These may take several forms, but the following types of comments are good examples:

- Health
- Air
- News
- Food (without mentioning anything else)
- Obesity
- Diabetes (or something like “my dad was a diabetic”)
- Wondering what the whole story was going to be about
- She’s 41
- I have been in her place before
- Wow, Michelle has a similar story to my. I am a type 2 diabetic.
- Anger
- I wonder what the study is about
- Michelle's picture was distracting
- David
- Events of the day
- Who is Michele Wolfe? Why was she chosen to be put in the study?
- I would have liked to read this myself. It wouldn’t have taken as long.
- I wonder if they made any spelling mistakes
- Lost weight (NOTE – without any indication of how she lost it)
- Her losing weight (NOTE – without any indication of how she lost it)
- Diabetes is a really scary disease.

These types of statements do not refer to a cause or solution to obesity and thus should be coded 0 for all three of these coding decisions.

**Q1: Does it mention an EXTERNAL CAUSE or SOLUTION for obesity?**

**1 = YES
0 = NO**

External causes and solutions refer to general or specific things that cause or could be used to reduce obesity. By external, we mean factors that are outside of an individual’s (or, in the case of childhood obesity, a parent’s) decisions, choices, behaviors, attributes or dispositions. External factors could include social (includes friends and any member of the family EXCEPT the parent of a child and NOT vice-versa), physical, economic, or policy factors. Any thought that mentions an external cause or solution for obesity should be coded as a “1,” regardless of whether or not the statement agrees or disagrees with that factor as an important cause or solution for obesity.

On occasion, a respondent will mention “stress,” “time,” or “education” without saying anything more about it, or with ambiguous additional information about it. While these factors could be attributable to external factors (stressful environments, busy work schedules, poor schools) or internal factors (poor stress or time management, failure to put in the effort to get an education), these factors should be coded as external if there is no additional information present to decide whether the statement is attributing stress, time or education to external or internal factors. If there is additional information that allows you to decide whether the respondent is referring to stress or time as internal or external, you should use this information and code accordingly.

Any use of the terms “excuses” or “complaining” should be coded as external because the excuses being referred to in the context of this story are almost certain to be external.

Any reference to the NDA (directly or indirectly), “financial pressures,” “poverty” or the “community” should also be coded as external.

**Examples:**

In the examples below, all of which should be coded as “1” here, the words or phrases with underlines are those that were used to classify these statements as external.

1. Her dangerous neighborhood leads to her lack of exercise
2. Low income should not affect what health benefits are available to you
3. I agree that it is at times easier and less expensive to buy staple foods or junk than to buy fruits and veggies
4. NDA provides an opportunity for better choices
5. Of course convenience foods are always cheaper—that’s a major problem with families I work with doing home health care
6. Creating walking paths should be done in more communities
7. Getting out in the neighborhood is hard
8. I feel it is a constant struggle to keep in shape or not be overweight in our overindulgent society
9. The food industry has a cap on it because humans only require a certain number of calories per day. In order to grow economically they must convince people to buy more food than they need
10. Cheap and easy food choices can replace healthy eating habits
11. Everyone is busy and has a hard time finding time to exercise
12. Who funds the NDA?
13. Packaged pasta and rice can make a lot of meals
14. Michelle is just making excuses;
15. Stop complaining

**Q2: Does it mention an INTERNAL and CONTROLLABLE CAUSE or SOLUTION for obesity?**

**1 = YES
0 = NO**

Internal and controllable causes and solutions refer to general or specific things that cause or could be used to reduce obesity. By internal, we mean factors that are within an individual’s (or, in the case of childhood obesity, a parent’s) decisions, choices, behaviors, attributes or dispositions. By controllable, we mean to exclude factors like genetic predispositions, biological factors or illnesses that are not caused by obesity. Any thought that mentions an internal and controllable cause or solution for obesity should be coded as a “1,” regardless of whether or not the statement agrees or disagrees with that factor as an important cause or solution for obesity. One useful strategy here is to look for verbs that describe individual actions – “tried” to lose weight; “walked” to the store; “engage in regular physical activity.”

Any use of the terms “lifestyle,” "eat" or "eating" “diet,” “exercise,” "choice," or "habit" should be coded as internal.

On occasion, a respondent will mention “diet-pills,” “weight-pills” or “knowledge (or knowing what is good or bad to eat)” without saying anything more about it. While these factors could be attributable to external factors (cost, aggressive marketing, poor education) or internal factors (laziness, lack of willpower, wanting the easy way out, failure to put in the effort to gain that knowledge), these factors should be coded as internal and controllable if there is no additional information present to decide whether the statement is attributing diet or exercise to external or internal factors. If there is additional information that allows you to decide whether the respondent is referring to diet or exercise as internal or external, you should use this information and code accordingly.

**Examples:**

In the examples below, the words or phrases in italics are those that were used to classify these statements as containing information about internal and controllable causes.

1. Her dangerous neighborhood leads to *her lack of exercise*
2. I agree that it is at times easier and less expensive *to buy staple foods or junk than to buy fruits and veggies.*
3. NDA provides an opportunity for *better choices*
4. *Getting out in the neighborhood* is hard
5. I feel it *is a constant struggle* *to keep in shape* or not be overweight in our overindulgent society
6. The food industry has a cap on it because humans only require a certain number of calories per day. In order to grow economically they must *convince people to* *buy more food* than they need.
7. Environment is essential *to healthy choices*
8. Cheap and easy food choices can replace *healthy eating habits*
9. *Eat more vegetables than meat*
10. *I eat in a very similar way with maybe a few healthier choices*
11. *You have to really try hard to change your eating habits*
12. *Vegetables can be easily grown* and more beneficial in the long run
13. *Curable with intent to succeed*
14. *I’m happy that she was able to find a better alternative to eating healthy*
15. Everyone is busy and *has a hard time finding time to exercise*

**Q3: Does the thought COUNTER, NEGATE, LIMIT, or CAVEAT whether an EXTERNAL CAUSE or SOLUTION for obesity exists or should be implemented?**

**1 = YES
0 = NO**

This code is intended for cases where the respondent either (1) directly and clearly negates external cause without mentioning an internal one, or (b) uses the internal cause to counter, limit or caveat a clearly articulated external cause.

Any use of the terms “excuses” or “complaining” should be coded as countering external because the excuses being referred to in the context of this story are almost certain to be external, and by calling them excuses or complaining the respondent is countering or negating them.

**NOTE: These codes can only occur if Q1 “EXTERNAL” is coded as 1**, since a thought cannot counter, negate, limit, or caveat an external cause or solution if it doesn’t mention that cause or solution.

**Examples** of directly negating an external cause or solution without mentioning internal ones:

1. Community doesn’t matter
2. NDA didn’t help this community
3. Michelle is just making excuses
4. Healthy food doesn’t cost that much
5. If the neighborhood wasn’t safe before, why is it safe on the jogging/bike trails?
6. As nice as having supermarkets and trails build everywhere would be, is it really a viable option?

**Examples** of mentioning both but emphasizing the internal one or using the internal one to counter, limit or caveat the external cause:

1. *It’s not that hard to make the healthier choice* even in low income neighborhoods
2. *Resilient people will find a way to exercise* even in tough areas of town
3. *People should walk more* regardless of parking areas
4. It’s not the responsibility of the neighborhood , rather *the responsibility is placed on the individual through self-discipline*
5. *Exercise can still occur* in crime cities like the use of treadmills in the home and simple other exercises.
6. *To be healthy it is not necessary to eat* expenses food *or go to* the gym.
7. I think it is not the NDA responsibility to make her health better *it should be her own no one else’s*
8. It does not cost money *to walk and to purchase healthy food*.
9. I don’t believe that it’s much more expensive to *eat healthfully* compared to fast food although I can see the conflict with items like spaghetti
10. Michelle is just making excuses

**Variable Re-Coding Procedure for Thought Variables**

Each respondent had up to 3 thoughts. Each thought was coded 0 or 1 for three variables: external, internal, and counter (see coding instructions listed above). Thus, for each respondent, there are the following nine variables:

**external_1** (whether or not the first thought mentioned an EXTERNAL CAUSE or SOLUTION for obesity)

**external_2** (whether or not the second thought mentioned an EXTERNAL CAUSE or SOLUTION for obesity)

**external_3** (whether or not the third thought mentioned an EXTERNAL CAUSE or SOLUTION for obesity)

**internal_1** (whether or not the first thought mentioned an INTERNAL and CONTROLLABLE CAUSE or SOLUTION for obesity)

**internal_2** (whether or not the second thought mentioned an INTERNAL and CONTROLLABLE CAUSE or SOLUTION for obesity)

**internal_3** (whether or not the third thought mentioned an INTERNAL and CONTROLLABLE CAUSE or SOLUTION for obesity)

**counter_1** (whether or not the first thought COUNTER, NEGATE, LIMIT, or CAVEAT whether an EXTERNAL CAUSE or SOLUTION for obesity exists or should be implemented)

**counter_2** (whether or not the second thought COUNTER, NEGATE, LIMIT, or CAVEAT whether an EXTERNAL CAUSE or SOLUTION for obesity exists or should be implemented)

**counter_3** (whether or not the third thought COUNTER, NEGATE, LIMIT, or CAVEAT whether an EXTERNAL CAUSE or SOLUTION for obesity exists or should be implemented)

Next, we created four mutually exclusive variables for each thought, based on responses to these variables.

Counterarguing

**cargue_1** If counter_1 = 1, regardless of values on external_1 or internal_1

**cargue_2** If counter_2 = 1, regardless of values on external_2 or internal_2

**cargue_3** If counter_3 = 1, regardless of values on external_3 or internal_3

Counter-elaboration

**celab_1** If internal_1 = 1 & external_1 = 0 & counter_1 = 0

**celab_2** If internal_2 = 1 & external_2 = 0 & counter_2 = 0

**celab_3** If internal_3 = 1 & external_3 = 0 & counter_3 = 0

Simple elaboration

**selab_1** If internal_1 = 0 & external_1 = 1 & counter_1 = 0

**selab_2** If internal_2 = 0 & external_2 = 1 & counter_2 = 0

**selab_3** If internal_3 = 0 & external_3 = 1 & counter_3 = 0

Complex integration

**complex_1** If internal_1 = 1 & external_1 = 1 & counter_1 = 0

**complex_2** If internal_2 = 1 & external_2 = 1 & counter_2 = 0

**complex_3** If internal_3 = 1 & external_3 = 1 & counter_3 = 0

Combined variables – number of each type of thought

**cargue_t** cargue_1 + cargue_2 + cargue_3

**celab_t** celab_1 + celab_2 + celab_3

**selab_t** selab_1 + selab_2 + selab_3

**complex_t** complex_1 + complex_2 + complex_3

Combined variables – whether or not respondent had at least one of each thought

**cargue**  cargue_1=1 OR cargue_2=1 OR cargue_3=1; else cargue=0

**celab** celab_1=1 OR celab_2=1 OR celab _3=1; else celab =0

**selab** selab_1=1 OR selab_2=1 OR selab _3=1; else selab =0

**complex_t** complex_1=1 OR complex_2=1 OR complex_3=1; else complex =0
